# Semi-mechanistic efficacy model for PARP + ATR inhibitors—application to rucaparib and talazoparib in combination with gartisertib in breast cancer PDXs

**DOI:** 10.1038/s41416-024-02935-w

**Published:** 2025-01-28

**Authors:** Claire C. Villette, Nathalie Dupuy, Frances A. Brightman, Astrid Zimmermann, Floriane Lignet, Frank T. Zenke, Nadia Terranova, Jayaprakasam Bolleddula, Samer El Bawab, Christophe Chassagnole

**Affiliations:** 1 Physiomics PLC, Abingdon, UK; 2 Merck Healthcare KGaA, Darmstadt, Germany; 3 EMD Serono Research & Development Institute, Inc., Billerica, MA USA; 4 Quantitative Pharmacology, Ares Trading S.A. (An Affiliate of Merck KGaA, Darmstadt, Germany), Lausanne, Switzerland

**Keywords:** Pharmacodynamics, Pharmacokinetics, Computational models, Predictive medicine

## Abstract

**Background:**

Promising cancer treatments, such as DDR inhibitors, are often challenged by the heterogeneity of responses in clinical trials. The present work aimed to build a computational framework to address those challenges.

**Methods:**

A semi-mechanistic pharmacokinetic-pharmacodynamic model of tumour growth inhibition was developed to investigate the efficacy of PARP and ATR inhibitors as monotherapies, and in combination. Key features of the DNA damage response were incorporated into the model to allow the emergence of synthetic lethality, including redundant DNA repair pathways that may be impaired due to genetic mutations, and due to PARP and ATR inhibition. Model parameters were calibrated using preclinical in vivo data for PARP inhibitors rucaparib and talazoparib and the ATR inhibitor gartisertib.

**Results:**

The model successfully captured the monotherapy efficacies of rucaparib and talazoparib, as well as the combination efficacy with gartisertib. The model was evaluated against multiple tumour xenografts with diverse genetic backgrounds and was able to capture the observed heterogeneity of response profiles.

**Conclusions:**

By enabling simulation of in vivo tumour growth inhibition with PARP and ATR inhibitors for specific tumour types, the model provides a rational approach to support the optimisation of dosing regimens to stratified populations.

## Background

Heterogenous treatment effect is a significant challenge in oncology drug development. Some tumours do not respond to a particular treatment, while others initially respond but ultimately develop resistance. Strategies to overcome these limitations include identification of genetic variants that can be used to stratify clinical outcomes, and thereby tailor treatment to specific populations. Alternatively, combination therapies can be deployed to overcome single agent failure. However, such approaches may require more extensive in vivo preclinical experiments to define the dose and dosing regimen in clinical trial design. Computational simulation using mathematical models, such as pharmacokinetic-pharmacodynamic (PK-PD) modelling, can address some of these challenges by integrating in vitro, in vivo, preclinical and clinical data, in order to explore underlying mechanisms of action and dose-response relationships for cancer treatments [1–7].

One therapeutic strategy that has gained significant attention aims to exploit the vulnerabilities of cancer cells arising from their genomic instability [8], by targeting the DNA damage response (DDR) machinery. Poly (ADP-ribose) polymerase (PARP) inhibitors are the first DDR targeted agents to have been successfully applied clinically for the treatment of various cancers. Four PARP inhibitors—olaparib, rucaparib, niraparib and talazoparib—are currently approved by the US Food and Drug Administration and the European Medicines Agency (EMA) for the treatment of cancers. PARP enzymes play a key role in the detection and repair of DNA damage in the form of single-strand breaks (SSB) and double-strand breaks (DSB), as well as replication fork damage [9–11]. PARP inhibitors not only abrogate the catalytic activity of PARP, but also prevent dissociation of PARP from DNA, thus blocking access to other repair mechanisms. The latter, PARP-trapping effect, is believed to be the main underlying mechanism of cytotoxicity of the four approved PARP inhibitors when used as monotherapy, as evidence suggests that PARP-DNA complexes are more deleterious than the absence of PARP [12]. However, normal cells with functional DDR are generally able to overcome the action of PARP inhibitors since they have redundant pathways to signal and initiate the repair of DNA lesions. On the other hand, PARP inhibition becomes critical when cells cannot activate alternative DNA repair mechanisms due to genetic defects in DDR. The strength of PARP inhibitors as single agents therefore lies in this principle of synthetic lethality. Thus, PARP inhibition has been shown in preclinical studies to be particularly effective in *BRCA*-mutant models [13, 14], which demonstrate homologous recombination (HR) deficiency (HRD). The association between PARP single-agent efficacy and *BRCA* mutation and, to a lesser extent, *BRCA*-wild type with HRD, has also been observed in clinical trials in ovarian cancer [15–20]. In accordance with this, PARP inhibitors have shown anti-tumour activity in advanced breast cancer with germline *BRCA* mutations [21, 22].

However, PARP inhibitors have limited application in clinical practice as single agents. *BRCA* mutations are infrequent in breast and ovarian cancers and ranged from 10 to 15% cases. HRD is more common in ovarian cancers, but current HRD biomarker testing fails to reliably identify patients whose tumours do not respond to PARP inhibitors in non-*BRCA* mutation groups [23]. Furthermore, most responders eventually develop resistance to treatment. Hence, alternative combination strategies with PARP inhibitors are being explored to extend therapeutic effect, broaden the range of indications and target a wider population [24]. One therapeutic option consists of creating a synergistic combination using a PARP inhibitor with other agents that compromise complementary DDR pathways. Potential candidates for such combination therapy are inhibitors of the ataxia telangiectasia and Rad3-related (ATR) protein kinase, a central regulator of the DDR [25–27]. ATR is essential for resolving DNA replication stress [25–27]. Stalled replication forks generate single-stranded DNA (ssDNA) segments where ATR is recruited to prevent fork collapse. Likewise, ATR becomes activated by exposed ssDNA ends that arise following DNA end resection during DSB repair. The kinase further plays a role in DSB repair as ATR signalling overlaps with the signalling of ataxia telangiectasia mutated (ATM) kinase in response to DSBs. In addition, ATR is critical for delaying cell-cycle progression when DNA damage is detected, including intra-S-phase and G2/M cell-cycle checkpoints, thus providing more time for DNA repair, and preventing damaged cells from entering mitosis. The combination of ATR and PARP inhibitors has shown promising results in preclinical studies [28–31], and this combination is now being assessed in multiple phase I/II clinical trials [32–34]. However, the clinical outcomes thus far have been mixed, and hence there is a clear need for methodologies that can better inform the development of DDR combination therapies.

This paper describes a semi-mechanistic PK-PD model that captures the in vivo (preclinical) tumour growth inhibition (TGI) with PARP and ATR inhibitors. The model represents key features of the roles of PARP and ATR in DDR pathway, allowing synthetic lethality to be induced by PARP inhibitors and increased replication fork stalling, double-strand breaks and apoptosis in combination with an ATR inhibitor. The model is calibrated with data generated in a *BRCA*-wild type HRD positive patient-derived xenograft (PDX) tumour model for PARP inhibitors rucaparib or talazoparib, the ATR inhibitor gartisertib [35, 36] and their combination. The work presented in this article also shows the model’s ability to capture the breadth of tumour responses across a PDX panel with various tumour characteristics.

## Methods

### Model overview

**Fig. 1:**
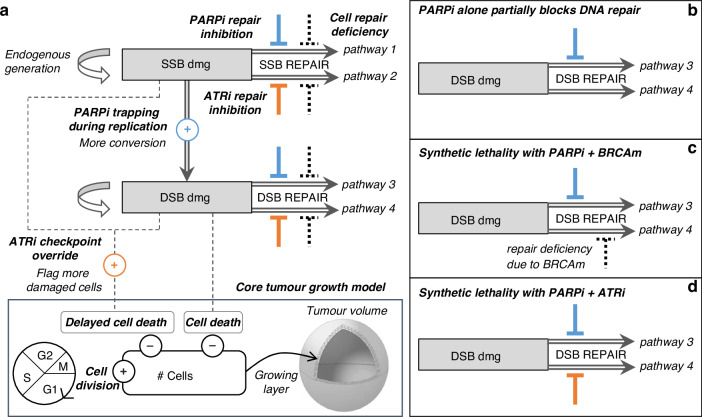
Schematic of the model structure showing PARP inhibitor effects (PARPi, in blue) and ATR inhibitor effects (ATRi, in orange). **a** Overall DDR model coupled to the core cell-cycle model. **b** PARPi monotherapy effect on cancer cell with fully competent DDR system: response will be poor due to damage repair performed by back-up pathway. **c** PARPi monotherapy effect on cancer cell with deficient back-up pathway (e.g. HR deficiency): response will be strong as both repair pathways are impaired. **d** PARPi + ATRi combination effect on cancer cell with fully competent DDR system: response will be strong as both repair pathways are impaired.

A PK-PD model has been developed to investigate the effects of PARP and ATR inhibitors on tumour growth as a function of drug concentration in plasma. The core PD model [3, 4, 6, 7] is a mathematical model of a virtual tumour with mechanistic features of the cell cycle, allowing simulation of the dynamics of tumour growth and the action of a given anti-cancer treatment. The tumour is assumed to have an outer layer of proliferating cells and an inner core that is quiescent or necrotic [2, 37–43]. A detailed description is provided in Supplementary Methods, including a comparison with Simeoni tumour growth model [44]. There are two sets of PD parameters, one set describing the mechanism of action of the drugs, and another set specific to tumour cell characteristics. The PD model was extended with an abstracted representation of the DDR, to allow the emergence of (i) synthetic lethality induced by PARP inhibition with inherent cell DNA repair deficiency and (ii) synergistic combination with the dual inhibition of PARP and ATR pathways. A schematic of the PD modelling framework is presented in Fig. 1.

The model outputs the tumour volume over time (assumed spherical), which can then be compared with TGI data in mice bearing triple-negative breast cancer (TNBC) PDXs (Table 2 and Supplementary Methods 8).

### DDR mathematical modelling

The features of the DDR model and its interaction with the tumour growth model are presented in Fig. 1. Parameter indices *p* and *a* refer to PARP and ATR inhibitors, respectively.

The DDR model incorporates two types of DNA damage, SSB and DSB. Equations 1 and 2 describe their evolution over time. Endogenous SSB and DSB damage are generated at fixed rates *r*_*ssb*_ and *r*_*dsb*_ and repaired at drug-modulated variable rates *R*_*ssb*_*(t)* and *R*_*dsb*_*(t)*. SSB damage is converted into DSB damage at rate *R*_*conv*_*(t)* in replication phase S.

1 $$\frac{{dSSB}}{{dt}}\left(t\right)={r}_{{ssb}}-{R}_{{conv}}^{\left({Sphase}\right)}\left(t\right)* {SSB}\left(t\right)-{R}_{{ssb}}\left(t\right)* {SSB}\left(t\right)$$

2 $$\frac{{dDSB}}{{dt}}\left(t\right)={r}_{{dsb}}+{R}_{{conv}}^{({Sphase})}\left(t\right)* {SSB}\left(t\right)-{R}_{{dsb}}\left(t\right)* {DSB}\left(t\right)$$

A proportion of SSB is transformed into DSB at the baseline conversion rate ${r}_{{conv}}$. PARP inhibitor will disrupt the repair of replication fork damage, and this effect *I*_*p_fork*_*(t)* triggers an acceleration of SSB conversion into DSB (Eq. 3).

3 $${R}_{{conv}}^{({Sphase})}\left(t\right)=\min \left(1,{r}_{{conv}}* \left(1+{I}_{p{\_}{fork}}\left(t\right)\right)\right)$$

SSB and DSB baseline repair rates *rep*_*ssb*_ and *rep*_*dsb*_ are modulated to reflect the disruption of DNA repair pathways associated with specific tumour models (Eqs. 4 and 5). The model assumes two parallel repair pathways per type of DNA damage, one mediated by PARP and the other mediated by ATR. Therefore, the inhibitory agents affect complementary repair pathways for SSB and DSB: PARP inhibition impairs pathways 1 (*I*_*p_ssb*_*(t)*) and 3 (*I*_*p_dsb*_*(t)*), while ATR inhibition impairs pathways 2 (*I*_*a_ssb*_*(t)*) and 4 (*I*_*a_dsb*_*(t)*). The inhibitory effect on SSB repair ${I}_{{a\_ssb}}(t)$ occurs in phase S to represent the disruption of ATR’s role in response to replication stress.

All four repair pathways are susceptible to specific impairment dependent on the genetic background of the tumour, modelled by four deficiency parameters, *def*_*1*_, *def*_*2*_, *def*_*3*_ and *def*_*4*_ (Eqs. 4 and 5). The value of these parameters ranges from 0 (fully functional) to 1 (total loss). The parameter *def*_*4*_ is associated with HR repair of DSBs. While HR is active during the S/G2 phases [45], HR deficiency is modelled throughout the entire cell cycle. This is justified by the notion that cells compensate for HR deficiency by activating alternative, error-prone repair pathways [9, 46], contributing to genomic instability. The other deficiency parameters (def1, def2 and def3) provide flexibility in capturing the heterogeneity observed in tumour responses, representing potential impairments in other DDR pathways, such as those resulting from ARID1A or ATM mutations.

4 $${R}_{{ssb}}\left(t\right)={{rep}}_{{ssb}}* \left(\left(1-{{def}}_{1}\right)* \frac{{e}^{-{I}_{{p}_{{ssb}}}\left(t\right)}}{1+{e}^{-{I}_{{p}_{{ssb}}}\left(t\right)}}+\left(1-{{def}}_{2}\right)* \frac{{e}^{-{I}_{{a}\_{{ssb}}}\left(t\right)}}{1+{e}^{-{I}_{{a}\_{{ssb}}}\left(t\right)}}\right)$$

5 $${R}_{{dsb}}\left(t\right)={{rep}}_{{dsb}}* \left(\left(1-{{def}}_{3}\right)* \frac{{e}^{-{I}_{{p}\_{{dsb}}}\left(t\right)}}{1+{e}^{-{I}_{{p}\_{{dsb}}}\left(t\right)}}+\left(1-{{def}}_{4}\right)* \frac{{e}^{-{I}_{{a}\_{{dsb}}}\left(t\right)}}{1+{e}^{-{I}_{{a}\_{{dsb}}}\left(t\right)}}\right)$$

A proportion of cells will undergo immediate cell death due to DSB (Eq. 6), which is cytotoxic, and the rate of DSB-induced cell death ${r}_{{kill}}^{({phase})}$ is phase dependent.

6 $${R}_{{kill}}\left(t\right)={r}_{{kill}}^{({phase})}* {DSB}\left(t\right)$$

A proportion of damaged cells will be sent to delayed cell death (Eq. 7), wherein cells continue to divide but eventually die after a few generations. The rate of delayed cell death ${r}_{{delay\_kill}}$ will be increased by the effect ${O}_{a}(t)$ induced by ATR inhibition. This effect reflects the override of ATR dependent cell-cycle checkpoints that would normally prevent damaged cells proceeding through the cell cycle. The override effect occurs in phases S to M.

7 $${R}_{{delay}{\_}{kill}}\left(t\right)=\left({r}_{{delay}{\_}{kill}}+{O}_{a}\left(t\right)\right)* \left(0.2* {SSB}\left(t\right)+{DSB}\left(t\right)\right)$$

The calculations of the number of live cancer cells in the outer proliferating shell, as well the tumour volume, are detailed in Supplementary Methods 4–6.

All drug effects $X(t)$ cited above—repair inhibitions ${I}_{{drug}}\left(t\right)$; and checkpoint override ${O}_{a}\left(t\right)$—take the general form of Eq. 8, as a function of ${C}_{{drug}}\left(t\right)$, the plasma concentration of the drug associated to that effect; ${u}_{x}$, the unit potency of the effect; and ${{Cl}}_{x}$, the clearance of the effect. Equation 8 for the drug’s effects allowed for temporal dissociation between the drug’s PK and PD, enabling sustained effect even after the drug had washed out with intermittent regimens. Biologically, this could reflect multiple complex interactions contributing to the overall effect, such as biological degradation, protein synthesis and the turnover of DDR molecules.

8 $$\frac{{dX}}{{dt}}\left(t\right)={u}_{x}* {C}_{{drug}}\left(t\right)-{{Cl}}_{x}* X(t)$$

The concentrations over time of the inhibitory agents are computed using plasma PK models described in Supplementary Methods 7. The drug protein binding and penetration in tumour tissues are captured implicitly by the model parameters.

### Calibration of the PD model parameters

PD model parameters that were kept constant across all simulations, regardless of the tumour model, are provided in Table S5. Drug-specific and tumour/experiment-specific parameters are listed in Table 1 and Table S6.

**Table 1:** Parameters of the DDR model calibrated for preclinical studies 1–4 in HBCx-9.

Parameter	Value for HBCx-9 in studies 1–4				Description
	1	2	3	4	
${t}_{{doub}}$	27	31	34	33	Cell doubling time (h)
${r}_{{ssb}}$	0.0005				Rate of endogenous generation of SSB damage (DAU/h)
${{def}}_{1}$	0	0	0	0	Repair deficiency for pathway 1 (SSB, PARP-mediated)
${{def}}_{3}$	0	0	0	0	Repair deficiency for pathway 3 (DSB, PARP-mediated)
${{def}}_{2}$	0	0	0	0	Repair deficiency for pathway 2 (SSB, ATR-mediated)
${{def}}_{4}$	20%	20%	10%	30%	Repair deficiency for pathway 4 (DSB, ATR/HR-mediated)
**Parameter**	**Value for gartisertib**				**Description**
${i}_{{a\_ssb}}$	7				Unit ATRi inhibitory effect on pathway 2 (/(mg/L)/h)
${i}_{{a\_dsb}}$	${i}_{{a\_ssb}}/2$				Unit ATRi inhibitory effect on pathway 4 (/(mg/L)/h)
${o}_{a}$	20				Unit ATRi checkpoint override effect (/DAU/h^2^/(mg/L))
${{cl}}_{a}$	0.15				Rate of clearance of ATRi effects (/h)
**Parameter**	**Value for rucaparib**		**Value for talazoparib**		**Description**
${i}_{{p\_ssb}}$	5		100		Unit PARPi inhibitory effect on pathway 1 (/(mg/L)/h)
${i}_{{p\_dsb}}$	${i}_{{p\_ssb}}$		${i}_{{p\_ssb}}$		Unit PARPi inhibitory effect on pathway 3 (/(mg/L)/h)
${i}_{{p\_fork}}$	0.5		170		Unit PARPi inhibitory effect on replication fork damage (/(mg/L)/h)
${{cl}}_{p}$	0.15		0.15		Rate of clearance of PARPi SSB/DSB repair inhibition effect (/h)
${{cl}}_{{p\_fork}}$	0.45		0.45		Rate of clearance of PARPi replication fork damage repair inhibition effect (/h)

DAU = arbitrary unit of DNA damage. PD model parameters that are fixed across all simulations are provided in Table S5.

Mouse TGI data with monotherapy and combination treatment arms were used to calibrate the DDR model parameters (Table 2). Four datasets (studies 1–4) were used to calibrate the parameters associated with the biology of HBCx-9 PDX and the mechanism of action of gartisertib. Of these four datasets, two were used to calibrate the parameters associated with rucaparib (studies 1 and 2) and two were used to calibrate those for talazoparib (studies 3 and 4).

**Table 2:** Summary of the mouse TGI data used in this study.

Study	PDX	PARP inhibitor (PARPi)	ATR inhibitor (ATRi) gartisertib	Treatment arms
1 *N* = 7	HBCx-9	rucaparib 50, 100 mg/kg qd x35 days, BID x35 days	3 mg/kg qd x35 days 3 mg/kg (1won/1woff)x2 + 1won	1x PARPi monotherapy 1x ATRi monotherapy 5x combination
2 *N* = 7	HBCx-9	rucaparib 50 mg/kg qd x28 days	10, 20 mg/kg once weekly x4 5, 10 mg/kg twice weekly x4 1, 3 mg/kg qd x28	1x PARPi monotherapy 2x ATRi monotherapy 6x combination
3 *N* = 8	HBCx-9	talazoparib 0.15 mg/kg BID x28 days	10, 20 mg/kg once weekly x4 5, 10 mg/kg twice weekly x4 1, 3 mg/kg qd x28 days 3 mg/kg qd x7 days	1x PARPi monotherapy 2x ATRi monotherapy 7x combination
4 *N* = 9	HBCx-9	talazoparib 0.15 mg/kg BID x6 or x8 weeks	20 mg/kg once weekly x6 20 mg/kg twice weekly x6 (*) 20 mg/kg three times a week x6 (*) (*) Dose reduced to 10 mg/kg from Day 13	1x PARPi monotherapy 1x ATRi monotherapy 3x combination
PDX panel *N* = 3	9 TNBC PDXs (*)	talazoparib 0.3 mg/kg qd x28	10, 20 mg/kg twice weekly x4	1x PARPi monotherapy 1x ATRi monotherapy 1x combination

*N* = number of mice per arm at the start of treatment for each study. Additional details, deviations and full list of PDXs(*) in Supplementary Methods 8.

To reflect the consistency of the agents’ mechanisms of action, the parameters associated with rucaparib, talazoparib and gartisertib effects were fixed across studies (Table 1). Similarly, most parameters associated with the biology of HBCx-9 were fixed across all four studies carried out in this tumour model (Table S5). Cell doubling time (*t*_*doub*_) and the repair deficiencies (*def*_*1-4*_) were allowed to vary across datasets, to accommodate the measurable differences observed in vehicle and monotherapy arms across the experiments (Table 1). For the PDX panel (Table S6), one dataset per tumour model was used to calibrate a subset of parameters associated with tumour biology: cell doubling time (*t*_*doub*_); repair deficiencies (*def*_*1-4*_); and endogenous SSB generation (*r*_*ssb*_).

The calibration was performed through a qualitative optimisation approach, whereby parameter values were manually and iteratively changed until the model simulations closely matched the TGI time series. Calibration was carried out considering the relatively small number of animals and the large variability that can be observed for a given treatment arm; parameter consistency was preferable to overfitting.

Most parameters represent an abstraction of the biology and thus do not have direct measurable biological markers. However, where possible, constraints informed by known biology were applied. The sources and rationale behind parameter values that were fixed across all simulations are provided in Table S5. For model simplicity, the PARP repair-inhibition effect on both SSB and DSB damage was fixed to the same value. For the ATR repair-inhibition effect, the impact on DSB was set to half the impact on SSB. This assumption was made to reflect the essential role played by ATR in the repair of replication-associated DNA damage (here modelled as SSB repair), while it was assumed that ATR inhibition would not fully disrupt the DSB repair pathway, as other signalling pathways, such as ATM (not incorporated in the model), would mitigate the inhibition [27]. For the repair deficiencies, the calibration process primarily focused on the *def*_*4*_ parameter related to HRD. As a result, *def*_*1*_*, def*_*2*_ and *def*_*3*_, were set to zero and only adjusted when necessary to capture the data.

## Results

### The model successfully captures the synergistic combination induced by rucaparib and gartisertib in HBCx-9

**Fig. 2:**
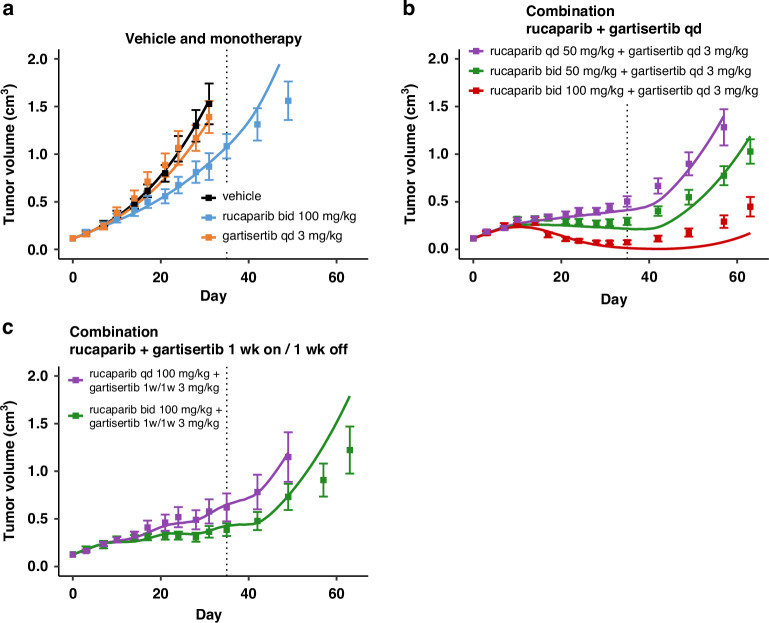
Model simulations overlaid with the TGI measured for various regimens in experimental study 1 in HBCx-9 tumour model. Treatment starts at Day 1, last dosing at Day 35 (dotted line). Solid line: simulation. Markers and error bars: data, mean tumour volume ± SEM. Data for each arm are shown when at least 6 out of 7 mice are still in the experiment. The arms are split across panels for clarity: vehicle and monotherapy (**a**), and combination (**b**,** c**). Rucaparib is given once (qd) or twice (bid) a day x35 days at the specified dose. Gartisertib is given once a day (qd, **b**) x35 days, or 1 week on 1 week off (1w1w, **c**) for 5 weeks total, at the specified dose.

**Fig. 3:**
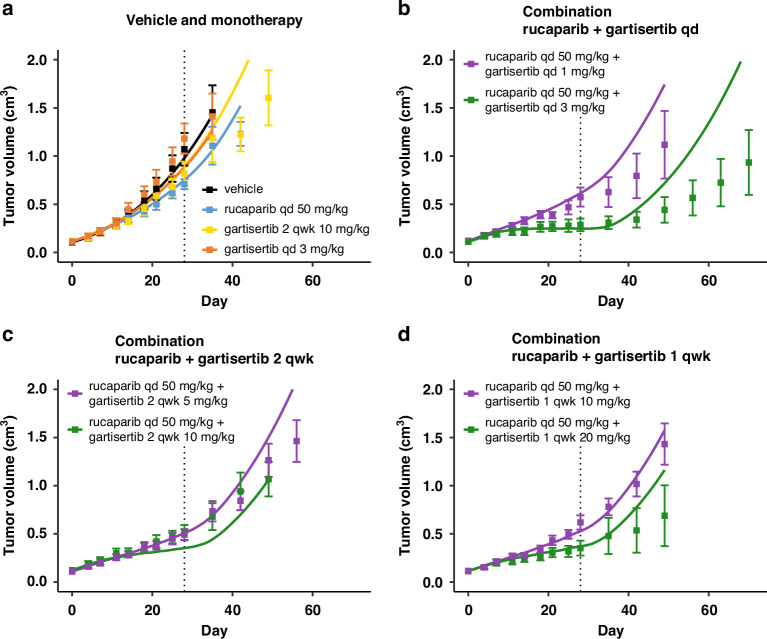
Model simulations overlaid with the TGI measured for various regimens in experimental study 2 in HBCx-9 tumour model. Treatment starts at Day 1, last dosing at Day 28 (dotted line). Solid line: simulation. Markers and error bars: data, mean tumour volume ± SEM. Data for each arm are shown when at least 6 out of 7 mice are still in the experiment. The arms are split across panels for clarity: vehicle and monotherapy (**a**), and combination (**b-d**). In all PARPi treated arms rucaparib is given daily at 50 mg/kg. Gartisertib is given once a day (qd, **b**) x28 days, twice a week (2 qwk, **c**) or once a week (1 qwk, **d**) x4 weeks, at the specified dose.

The model captures the dose response of the various arms in studies 1 and 2 with rucaparib and gartisertib, for both monotherapies and combination in the HBCx-9 PDX (Figs. 2 and 3). In the two calibration datasets, the monotherapies show little or no anti-tumour activity (Figs. 2a and 3a), despite the tumour model being HRD positive.

For the calibration of HBCx-9 cell-specific parameters, it was assumed that SSB damage repair in both pathways 1 and 2 was fully competent. Hence, even if PARP inhibitor as single agent completely supresses the SSB repair pathway 1, the total repair disruption is capped at 50%, as the parallel ATR-mediated repair pathway 2 is always intact (Eq. 4). On the other hand, the calibration introduced a repair deficiency for DSB damage in the ATR/HR-mediated pathway 4 (*def*_*4*_ in Table 1). Thus, contrary to SSB, when treated with PARP inhibitor alone, the total repair inhibition for DSB damage is the sum of the two disruptions from each pathway (Eq. 5). The baseline disruption of DSB repair due to innate deficiencies is relatively low (*def*_*4*_ = 20%; Table 1), limiting the extent of synthetic lethality when rucaparib is administered alone (Figs. 2a and 3a). Better efficacy is achieved when combining rucaparib with the ATR inhibitor gartisertib (panels b–d in Figs. 2 and 3). Synergistic combination efficacy arises as the two agents simultaneously target complementary DDR pathways, as illustrated in Fig. 1. All three effects associated with ATR inhibition included in the DDR model—SSB repair inhibition, DSB repair inhibition and checkpoint override—contribute to the final antitumor activity obtained with concurrent PARP inhibition (Fig. S2).

It should be noted that the model was primarily designed to capture the TGI effects during the treatment period. As a result, divergences between the model simulations and the mean experimental data may occur at later time points, after the final dose has been administered. However, it is important to consider the large standard errors in the TGI data at these later time points, driven by data drop-out as animals are sacrificed, as well as greater heterogeneity in tumour behaviour once the drug has washed out (see individual TGI profiles for Study 2 in Fig. S3A). The model generally falls within the observed variability and is represents some individual animal TGI profiles.

Furthermore, the model captures the mean effect reasonably well up to 1–2 weeks post-treatment and, in cases of response, up to about 3 weeks. This timeframe is reasonable considering the typical dosing holidays in intermittent regimens for PARPi and ATRi explored in clinical trials. The model also successfully captures the effects of ATRi regimens with a 1-week break (Figs. 2c, 3d), demonstrating its ability to simulate TGI under intermittent regimens, despite deviations observed post-treatment.

There is, however, a model divergence before the end of treatment for one combination arm in study 2 (Fig. 3c), which may be attributed to experimental variability causing data inconsistencies. Specifically, while tumour responses are consistent across all regimens with a total weekly dose of ~10 mg/kg for gartisertib (panels b, c and d in Fig. 3), a mismatch is observed with the ~20 mg/kg weekly dose between the experiment in panel C and the two other regimens in panels b and d.

Overall, the objective was to capture an average response across heterogeneous tumour profiles for a given tumour model, rather than fine-tuning each experimental arm, with a focus on capturing differences between genetic backgrounds. Nevertheless, the model could be further refined using population modelling to better account for inter-animal and experimental variabilities. As an illustration, two local sensitivity analyses were conducted by varying two key model parameters: first, cell doubling time (Fig. S3A), which accounts for a large portion of the observed variability within experimental arms; and second, the parameter related to HR deficiency (def4, Fig. S3B), which modulates responses to PARPi therapies. A population modelling approach would enable the fine-tuning of parameter sets to describe individual response profiles.

### The model successfully translates to the PARP inhibitor talazoparib in combination with gartisertib in the same tumour model HBCx-9

**Fig. 4:**
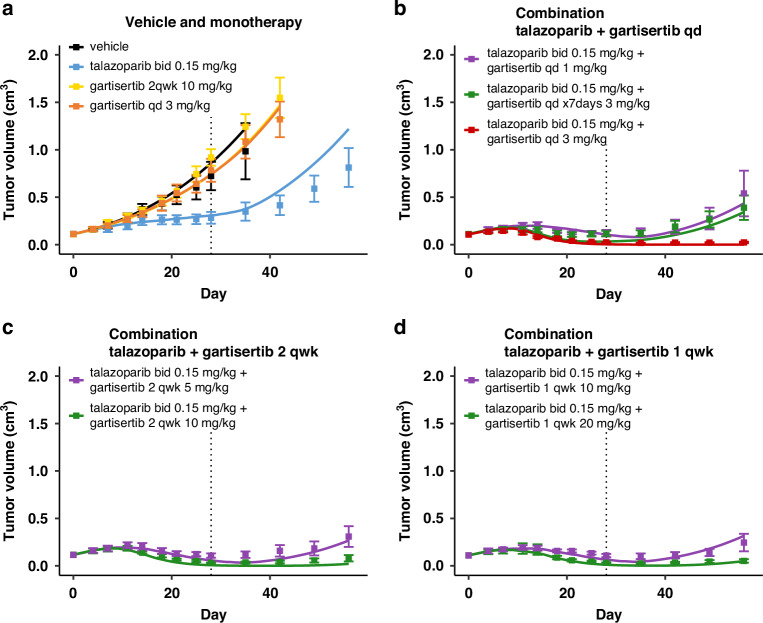
Model simulations overlaid with the TGI measured for various regimens in experimental study 3 in HBCx-9 tumour model. Treatment starts at Day 1, last dosing at Day 28 (dotted line). Solid line: simulation. Markers and error bars: data, mean tumour volume ± SEM. Data for each arm are shown when at least 6 out of 8 mice are still in the experiment. The arms are split across panels for clarity: vehicle and monotherapy (**a**), and combination (**b-d**). In all PARPi treated arms talazoparib is given twice a day at 0.15 mg/kg. Unless specified in the legend, gartisertib is given once a day (qd, **b**) x28 days, twice a week (2 qwk, **c**) or once a week (1 qwk, **d**) x4 weeks, at the specified dose.

The parameters describing the mechanism of action of PARP inhibitors were successfully recalibrated to capture the TGI effects of talazoparib and its combination with gartisertib (Fig. 4 and Fig. S4). Notably, the drug-effect parameter values of talazoparib in the model are approximately two orders of magnitude higher than those for rucaparib (Table 1). These calibrated values for PARP inhibitors align with their relative potencies reported in the literature, where talazoparib is also about two orders of magnitude more potent than rucaparib [47, 48].

To better compare the dose-response profiles of the two agents, PK-TGI simulations were conducted across a range of doses for both PARP inhibitors in HBCx-9 using the four tumour parameter sets calibrated from studies 1–4 (Fig. S5A, B, dark and light blue lines). The simulations confirm that, as a single agent, talazoparib achieves efficacy at much lower exposures—and doses—than rucaparib. These monotherapy simulations reveal that once tumour response is observed, the total level of SSB/DSB repair inhibition rapidly saturates and becomes equivalent between talazoparib and rucaparib at that point (Fig. S5E, F). The saturation level depends on repair deficiencies, with a greater impact on DSB repair pathways, which are inherently deficient in HBCx-9 (*def*_*4*_ in Table 1). Hence, as PARPi exposure increases, the primary driver of DSB accumulation in the model (Fig. S5C) is the accelerated degradation of SSBs into DSBs during the replication phase of the cell cycle (Fig. S5D).

The model simulations also suggest the extent to which PARPi doses can be reduced when combined with ATRi, while still achieving tumour shrinkage (Fig. S5B, red and yellow lines). The addition of gartisertib alongside PARP inhibition further compromises both DNA damage repair and fork stabilisation, and leads to increased delayed cell death as a result of cell-cycle checkpoint override (Fig. S5G). Collectively, these effects contribute to greater DSB accumulation (Fig. S5C).

Overall, the model simulations across the four calibrated versions of the HBCx-9 tumour model demonstrate that, despite parameter variations, the TGI predictions remain consistent. However, the slight differences observed offer valuable insights into the heterogeneity of the average response that may be expected for a given treatment in a specific tumour model.

### The model successfully captures heterogeneous TGI profiles across a TNBC PDX panel with diverse genetic backgrounds

With the drug models for talazoparib and gartisertib in HBCx-9 parameterised, the next step was to determine whether the calibrated mechanisms of action would apply to other tumour models. The aim was to assess the model’s ability to generate heterogeneous TGI responses to PARP and ATR inhibitors based on distinct tumour characteristics. To this end, a panel of 9 TNBC PDXs with multiple genetic backgrounds was investigated, grouped into three categories for simplicity: (i) *BRCA*-mutant; (ii) *BRCA*-wild type HRD positive, which includes HBCx-9; and (iii) HRD negative.

**Fig. 5:**
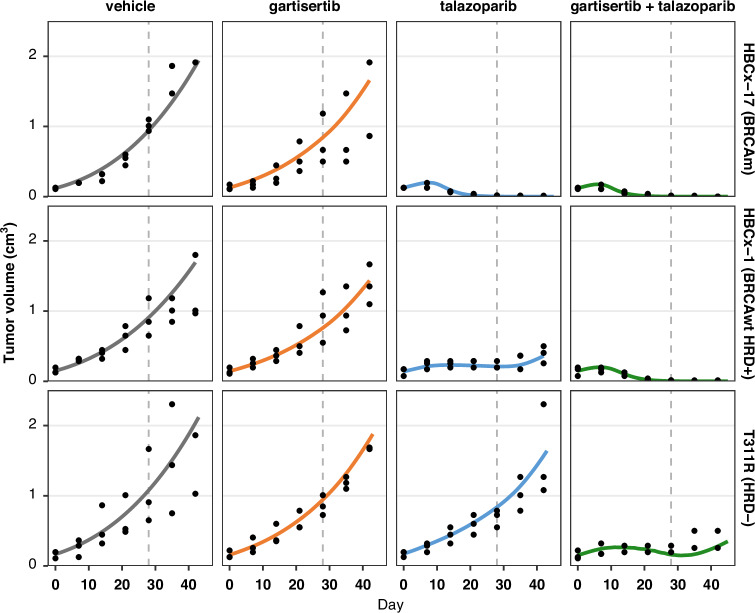
Model simulations overlaid with the TGI data for three studies in the TNBC PDX panel with different genetic backgrounds. Simulation (lines), data (black dots; *n* = 3 animals per arm at start of treatment). Treatment starts at Day 1, last dosing at Day 28. The four arms in each study are split in columns. Each row is a different PDX tumour model with specific genetic background. Treatment: talazoparib is given once a day (qd) at 0.3 mg/kg x28 days; gartisertib is given twice per week, x4 cycles, at doses of 10 mg/kg (HBCx-17, HBCx-1) or 20 mg/kg (T311R). Additional details are provided in Supplementary Methods 8.

A subset of cell-specific parameters was recalibrated for each study (Table S6), enabling the model to capture the diverse anti-tumour activities observed across both monotherapy and combination therapy experiments (Fig. 5 and Fig. S6). In all *BRCA*-mutant PDXs, talazoparib was highly effective as a single agent, with minimal added benefit when combined with gartisertib. In two of these calibrated tumour models, HBCx-10 and HBCx-17, the DSB repair via ATR/HR pathway 4 was significantly impaired (*def*_*4*_ > 80%), leading to high synergy with PARPi. In the third *BRCA*-mutant model, HBCx-22, talazoparib monotherapy did not result in complete tumour regression, unlike the other two PDXs, which is reflected by a lower deficiency parameter value.

In the *BRCA*-wild type HRD positive group, response profiles were heterogeneous. The HBCx-1 and HBCx-9 models were characterised by mid-range DSB repair deficiency in ATR/HR pathway 4, and the TGI effect was greater with talazoparib and gartisertib combination compared to the PARPi alone. Interestingly, the calibrated *def*_*4*_ parameter for HBCx-9 in this set of experiments was higher than in the original four studies (50% versus 10–30%), potentially reflecting further heterogeneity or experimental variability due to data sparsity. Conversely, HBCx-15 model displayed a high DSB repair deficiency in ATR/HR pathway 4, leading to complete tumour regression with talazoparib monotherapy. In this model, the introduction of a deficiency in the PARP-mediated DSB repair pathway (*def*_*3*_) was necessary to increase the efficacy of gartisertib monotherapy (Fig. S6).

Finally, the model captures the response patterns in the HRD negative group, where the combination therapy proved most effective, as talazoparib alone did not stabilise tumour growth. In this PDX group, the calibrated models had no repair deficiency in ATR/HR pathway 4 and for T311R and HBCx-30, reducing the endogenous SSB damage generation parameter was necessary to capture the data, consistent with a more competent DDR system.

## Discussion

### Novel preclinical PK-TGI framework for DDR agent efficacy across tumour models

This paper outlines a semi-mechanistic PK-PD model to simulate in vivo TGI, focusing on key features of the cell DDR and agents targeting specific DDR pathways. The application of the model has been illustrated with two PARP inhibitors, rucaparib and talazoparib and the ATR inhibitor gartisertib. The PD model deconvolutes the parameters describing the drug mechanism of action, which are fixed for a given drug, from those that reflect cancer cell characteristics, which are adjusted to modulate tumour response. By incorporating tumour *BRCA*/HRD biomarkers that have been shown to be associated with PARPi sensitivity in the clinic [49], the model was able to capture a spectrum of TGI response profiles for each treatment, depending upon innate DNA repair capability. For instance, with the same PARP inhibitor regimen, the model simulates both a lack of response in cell lines with competent DDR and synthetic lethality in HRD positive tumours. Furthermore, synergistic behaviour between PARP and ATR inhibitors arises as a natural consequence of combining the mathematical representations of their mechanisms of action, which impact complementary parts of the DDR machinery. Thus, model simulation of combination regimens did not require the introduction of additional ‘synergy’ factors, and neither did it require ‘scaling’ factors for different tumour models; instead, different levels of combination synergy were achieved through the interaction of drug and cell line parameters. This modelling approach, combined with a rich preclinical TGI dataset of talazoparib and gartisertib across multiple cell lines with varied mutational statuses, yielded a panel of PDX-specific DDR models that can be repurposed to predict the in vivo preclinical efficacy of other similar drugs. For example, using rucaparib parameters solely calibrated in the HBCx-9 tumour model, simulations of monotherapy and combination with gartisertib can be conducted in any tumour model within the PDX panel by applying the corresponding tumour-specific parameters. This modular approach enables the generation of dose-exposure-response curves (Fig. S5) for any drug in any calibrated tumour model, without requiring specific data on every drug-tumour combination. As a result, the model helps alleviate ethical and resource constraints by using extensive in silico experiments to guide the focus on the most relevant in vivo studies. In contrast, other PK-PD models use parameters that simultaneously capture both tumour characteristics and drug effects [44, 50], limiting their ability to explore tumour responses across multiple PDXs without specific data for each scenario.

### An abstract model of DDR mechanisms with potential for biomarkers refinement

The PD model presented in this work is a high-level abstraction of the most essential tumour characteristics and inhibitor mechanisms of action related to DDR, yet its parameters are intuitively linked to known biological processes. Within the context of quantitative systems pharmacology, this PD model relies on a relatively small number of parameters, considering its ability to capture tumour growth dynamics across multiple drugs, doses, schedules and xenograft models. Only four independent parameters for each PARP inhibitor and three for the ATR inhibitor gartisertib were necessary to robustly characterise the mechanism of action of the agents across various PDXs. Most parameters describing the cancer cell DDR could be fixed across tumour models (Table S5). Overall variability in treatment response could largely be accounted for by adjusting cell doubling time, while the DSB repair deficiency parameter associated with HR specifically modulated the response to PARPi treatments (Table 1 and S6). The remaining parameters allow flexibility for calibration, since other genetic sources of DDR functional impairment that have not been considered in the current model may impact tumour response. The only data required to calibrate the DDR parameters were in vivo TGI data and the *BRCA* and HRD characterisations of the PDXs. However, incorporating additional biomarkers—such as γH2AX (for DSBs), p-Chk1 (for DDR checkpoint activation) and RAD51 foci assays (for HR repair functionality)—could further refine the representation of drug synergy, saturation and timing effects and cancer cell sensitivity to PARP and ATR inhibitors, improving model predictivity for different dosing strategies in specific tumour types.

### Limitations of the current PK-PD model calibration

The consistency of parameters across a broad range of TGI datasets strengthens confidence in the model. However, caution is necessary when interpreting the current model due to several limitations in the data. Firstly, the calibration of the PDX tumour models with the talazoparib + gartisertib combination relied on sparse data, with only three animals per arm, which, given the variability observed in larger datasets, may not be sufficient to derive the true average TGI. Furthermore, no monotherapy arms with the ATRi gartisertib demonstrated significant anti-tumour activity in the available datasets, which may have limited the calibration of its mechanism of action. A more complete monotherapy dose-response TGI dataset for ATRi would enable a re-evaluation of its parameterisation. Lastly, while the current DDR model can simulate intermittent regimens, the calibration datasets included only up to 1 week of dosing breaks, and only for the ATRi. Further preclinical data with extended off-treatment periods would be valuable, especially given the importance of these regimens in clinical trials.

### Utility of the PK-PD model for clinical translation and optimising combination regimens

The model can guide the selection of regimens for clinical trials, where the challenge is to find combination regimens that are both effective and tolerable for specific patient populations. Indeed, combinations of DDR-targeting agents are often limited by overlapping toxicities, leading to the exploration of alternative clinical dosing strategies to improve tolerability [51]. However, for regimens with similar expected toxicity, combination therapies of small molecule inhibitors may not always outperform single-agent treatments in terms of efficacy [52]. Mixed results from phase II clinical trials involving the PARPi olaparib and ATRi ceralasertib highlight these challenges. In the VIOLETTE trial in TNBC (NCT03330847), no significant difference was observed between olaparib plus ceralasertib and olaparib monotherapy, irrespective of HR characterisation [32]. These results led to the withdrawal of the related DUETTE study in ovarian cancer (NCT04239014). In smaller cohorts of tens of patients with recurrent ovarian cancer, the CAPRI trial (NCT03462342) showed promising results in the platinum-sensitive HRD cohort with acquired PARPi resistance, but the combination did not meet expectations in the platinum-resistant group [33, 34]. The modelling framework presented here can help optimise regimens by comparing the relative efficacy of dosing strategies currently used in the clinic with others that are identified or predicted to be clinically tolerable. The current PK-TGI model can be translated to clinical settings by simulating PARPi + ATRi combination regimens, including intermittent and sequential schedules, using human PK data to adjust drug concentration dynamics. However, this approach relies on the critical assumption that the drug mechanisms and synergies observed in preclinical models will translate to humans. Importantly, each dosing regimen of PARPi and ATRi can be evaluated across diverse genetic backgrounds using calibrated preclinical PDX models. These model simulations can support the selection of the most appropriate regimens for specific populations, while the analysis of responding xenografts can provide valuable insights into tumour characteristics potentially linked to clinical anti-tumour activity. Finally, the model’s ability to quantify the heterogeneity in treatment responses to PARPi and ATRi can also inform statistical power calculations for clinical trial protocols, either in global populations or in stratified cohorts based on *BRCA* and HRD status.

### Potential extensions of the DDR Model for future applications

It is anticipated that the model can be readily extended to explore additional cancer therapeutic strategies involving the DDR. One extension could introduce acquired PARPi-resistance. For example, the functional recovery of initially deficient HR-mediated DNA repair and the increased ATR-CHK1 activity—both associated with the emergence of PARPi resistance [53, 54]—could be incorporated into the model by reducing the DSB repair deficiency value on pathway 4. A second extension could include synthetic lethal interactions with PARP and ATR inhibitors beyond *BRCA* loss and HRD, such as ARID1A [55, 56] and ATM mutations [10, 29, 57], which would further help identify tumours most likely to respond to specific treatments. A third extension could integrate the mechanisms of action of agents targeting different DDR pathways, such as ATM and DNA-PK inhibitors [36, 58], as well as DNA damage-inducing agents, such as chemotherapy or radiation [30, 59, 60], thus broadening the model’s application to a wider range of combination therapies.

To conclude, the computational framework presented here can be used as a tool for quantifying the relative benefits of various monotherapy or combination treatments that target DDR in characterised tumour types, and thereby enable a rational approach for selecting the right therapeutic regimen for the right population.

## Supplementary information

Supplementary Material

## Data Availability

Any requests for data by qualified scientific and medical researchers for legitimate research purposes will be subject to Merck’s (CrossRef Funder ID: 10.13039/100009945) Data Sharing Policy. All requests should be submitted in writing to Merck’s data sharing portal (https://www.merckgroup.com/en/research/our-approach-to-research-and-development/healthcare/clinical-trials/commitment-responsible-data-sharing.html). When Merck has a co-research, co-development, or co-marketing or co-promotion agreement, or when the product has been out-licensed, the responsibility for disclosure might be dependent on the agreement between parties. Under these circumstances, Merck will endeavour to gain agreement to share data in response to requests.

## Code availability

The code of the virtual tumour model can be made available through a commercial license.
