# Supplementary material for: Semi-mechanistic efficacy model for PARP + ATR inhibitors—application to rucaparib and talazoparib in combination with gartisertib in breast cancer PDXs
Source: Br J Cancer. 2025 Jan 28;132(5):481–91. doi: 10.1038/s41416-024-02935-w (PMC11876674; doi:10.1038/s41416-024-02935-w)
Supplement: Supplementary file 1 — Supplementary Material [file 41416_2024_2935_MOESM1_ESM.docx]

# **List of supplementary documents**

*List supplementary methods*

1. Tumour growth model
2. Tumour growth model justification
3. Cell population PD model
4. Tumour volume calculation
5. Cellular volume calculation
6. Cell number calculation
7. Pharmacokinetic models
8. Preclinical TGI data

*List supplementary tables*

- Table S1. PK model parameters for gartisertib.
- Table S2. PK model parameters for rucaparib camsylate.
- Table S3. PK model parameters for talazoparib.
- Table S4. Phase-specific processes during the cell cycle.
- Table S5: PD model parameters fixed across all simulations
- Table S6. Model parameters characterising the cancer cell line of the PDX panel.

*List supplementary figures*

- Figure S1. Tumour growth models comparison.
- Figure S2. Model simulation: in the DDR model, three effects due to ATR inhibition contribute to the synergistic combination with the PARP inhibitor rucaparib.
- Figure S3. Model simulation overlaid with the TGI of each animal in experimental study 2 in HBCx-9 tumour model, and parameter local sensitivity.
- Figure S4. Fourth data set for the calibration of drug parameters. Model simulations overlaid with the TGI measured for various regimens in experimental study 4 in HBCx-9 tumour model.
- Figure S5. Simulated dose-exposure-response of rucaparib and talazoparib in monotherapy and in combination with gartisertib in HBCx-9.
- Figure S6. PDX panel (continued). Model simulations overlaid with the TGI data for the other studies in the TNBC PDX panel with different genetic backgrounds.
- Figure S7. Preclinical PK model fitting for gartisertib.
- Figure S8. Preclinical PK model fitting for rucaparib.

# **Supplementary Methods**

## **Tumour growth model**

The tumour is assumed to have a spherical shape, with an outer layer of proliferating cells that drive tumour growth and an inner core that is quiescent or necrotic. Indeed, cells in the core of solid tumours have limited blood supply, and thus low oxygen levels (hypoxia) and insufficient delivery of nutrients. The growth model used in the present study is based on the work of Mayneord (1) who reported that the increase in diameter of untreated grafted tumours in rats followed a linear profile, which could be explained by the observation that only a thin shell surrounding the tumour was in a state of active division. The main assumption of the model is that the thickness of the outer proliferating layer must be smaller than the total tumour radius, which holds in the case of the xenograft models in this study since, by the start of the TGI experiments, the tumours have already grown to a size where they have formed a central core. In all model simulations, the maximum thickness of the outer growing layer was set to 0.25 mm (value derived from (2)), which is much smaller than the initial radius in every preclinical study (~3 mm). Tumour growth models based on similar principle have been used in other modelling work, including preclinical (3–6) as well as clinical (7,8) applications.

The linear growth model in radius was well suited to capture the observed data of untreated tumours for all experiments in this study. A detailed description and comparison of the present model with Simeoni model (9) is provided in ***Supplementary Methods 2: Tumour growth model justification.***

## **Tumour growth model justification**

The growth law model used in this model is based on the work of Mayneord (1), where they observed that only the outer rim of the tumour was viable and proliferating, and that the total tumour radius tended to increase linearly over time. There is a simple linear equation describing the relationship between the two observations:

|  | $R\left( t \right)= R\left( t=0 \right)+g*t$  $g=\frac{ln(2)}{t_{d}}*r_{shell}$ | (S2.1) |
| --- | --- | --- |

where $R$ is the total tumour radius and $g$ the growth rate of the tumour, $r_{shell}$ is the thickness of the outer layer, here a constant, and $t_{d}$ is the cell cycle length of the dividing cells in the shell. A derivation of the equation can be found here (4). This model is particularly relevant for *in vivo* xenograft experiments where, by the time of randomisation, the tumour has a defined structure with non-dividing core and proliferating shell (2). When fitting to the TGI data, $g$ is the single parameter to estimate. As the radius increases linearly, the tumour volume (sphere) increases cubically.

On the other hand, the Simeoni tumour growth model (9), a well-known model for PK/TGI modelling, describes the tumour growth from early phases of development. It assumes a first phase of exponential growth, followed by a linear growth in volume. The model is defined as an ODE:

|  | $\frac{dV}{dt}(t)=\frac{\lambda_{0}*V(t)}{\left[ 1+\left( \frac{\lambda_{0}}{\lambda_{1}}*V(t) \right)^{\Psi} \right]^{1/\Psi}}$ | (S2.2) |
| --- | --- | --- |

where $\Psi$ is a parameter fixed by the author to 20, and the two parameters $\lambda_{0}$ and $\lambda_{0}$ remain to be estimated to fit the TGI data.

Upon investigating the observed radius of untreated tumours (vehicle arms) in the TGI data, it was clear that the general trend showed a linear growth over time (see data examples in Figure S1A; grey lines with markers). For completeness, the Mayneord and Simeoni models were fitted to the vehicle arms of all preclinical experiments in this study (13 data sets). The fitting was done in R using the *minpack.lm* package. The objective function was to minimise the error between predicted and observed tumour radius for both models to allow for comparison.

A very important consideration is that animals with large tumours are sacrificed over time. Various methods exist to adjust automatic fitting processes to account for the missing animals at later time points; however, a simple approach was chosen here. The fitting was done only up to the time points where at least 70% of the animals remained (for experiments with only 3 animals per arm, all animals had to remain). The advantage of this approach was that it also allowed for an assessment of each model’s ability to predict tumour growth beyond the last time point of fitting. This was particularly evident in the experiment 'Study-4' in HBCx-9, shown in Figure S1A, which was fitted up to 32 days. The Mayneord model more accurately captured the tumour growth data beyond that time point. Overall, the Mayneord and Simeoni models had similar fitting errors (Figure S1C); however, the Mayneord model exhibited significantly less variability in parameter estimates (lower %RSE; Figure S1B). Consequently, the Mayneord representation of tumour growth was selected because it is simpler, effectively captures the general trend of the noisy data, and reflects the underlying biology of solid tumours (outer rim of dividing cells).

Finally, the model described in this work (designated as 'Virtual Tumour') is based on the same principle as the Mayneord model and approximates tumour growth as linear in radius due to the outer shell expansion (Figure S1A).

## **Cell population PD model**

The growth of the tumour outer shell is caused by the proliferation of dividing cells. These cells are assumed to have the same cell cycle length $t_{d}$ and the same initial volume at the start of G1 phase. Hence, when a cell divides at mitosis, it produces two daughter cells of equal volume.

The proliferating shell contains multiple cell sub-populations. Each sub-population tracks the evolution of its progeny over generations as the cells synchronously progress through the cell cycle and divide over time. The different sub-populations start the simulations from a different cell cycle phase (G1, S, G2 or M), with their starting position uniformly distributed over the cell cycle, but they all follow the same set of rules defined by the PD model.

The PD model consists of a set of ordinary differential equations describing progress through the cell cycle (10–12) and the DDR, described in the next method section. Some operations are phase specific as listed in Table S4. The model tracks the evolution of the number of live cancer cells in the shell over time, cells that may die due to DNA damage. A delayed cell-death course is also implemented, consistent with experimental observations of damage accumulation leading to cell death after several generations (13,14). The cell count is then converted into tumour volume at each time step. Details are provided in ***Supplementary Methods 4-6.***

The PD model was implemented and solved in MATLAB using a finite difference algorithm with 0.1 h time steps. Initial conditions reflect the steady state between DNA-damage generation and repair as well as the absence of drug effects.

## **Tumour volume calculation**

The tumour volume $V_{tumor}\left( t \right)$is computed incrementally after each simulation time step $dt$. As described in the main text method, the tumour is assumed to have a spherical shape comprising a **necrotic** **inner** **core**, where the cells are quiescent, and a **proliferating outer** **shell**, where the cancer cells progress through the cell cycle and divide.

The shell volume $V_{shell}$ grows at time $t+dt$ at rate $g_{shell}$:

|  | $\Delta V_{shell}\left( t+dt \right)=g_{shell}\left( t+dt \right)*V_{shell}\left( t \right)$ $V_{shell}\left( t+dt \right)=V_{shell}\left( t \right)+ \Delta V_{shell}\left( t+dt \right)$ | (S4.1) |
| --- | --- | --- |

The growth rate of the shell $g_{shell}$ is equal to the relative change in cellular volume $V_{cells.shell}$ (see ***Supplementary Methods 5: Cellular volume calculation***):

|  | $g_{shell}\left( t+dt \right)=\frac{V_{cells.shell}\left( t+dt \right)-V_{cells.shell}\left( t \right)}{V_{cells.shell}(t)}$ | (S4.2) |
| --- | --- | --- |

As the cancer cells divide, the shell thickens $\Delta V_{shell}\left( t+dt \right)>0$, and the total tumour volume $V_{tumour}$ grows:

|  | $V_{tumour}\left( t+dt \right)=V_{core}\left( t \right)+V_{shell}\left( t+dt \right)$ $=V_{core}\left( t \right)+V_{shell}\left( t \right)+ \Delta V_{shell}\left( t+dt \right)$ | (S4.3) |
| --- | --- | --- |

The thickness of the shell, however, cannot exceed a maximum thickness $r_{shell}^{MAX}$, as hypoxia and lack of nutrients lead to cell necrosis. The thickness of the outer proliferating layer is measured as follows:

|  | $r_{shell}\left( t+dt \right)=r_{tumour}\left( t+dt \right)-r_{core}\left( t \right)$ | (S4.4) |
| --- | --- | --- |

where $r_{tumour}$ and $r_{core}$ are computed from their respective volumes:

|  | $r_{tumour}\left( t+dt \right)=\left( \frac{3}{4\pi}V_{tumour}(t+dt) \right)^{1/3}$ $r_{core}\left( t \right)=\left( \frac{3}{4\pi}V_{core}(t) \right)^{1/3}$ | (S4.5) |
| --- | --- | --- |

- **If** $\boldsymbol{\Delta}\boldsymbol{V}_{\boldsymbol{shell}}\left( \boldsymbol{t+dt} \right)\boldsymbol{>0}\text{and }\boldsymbol{r}_{\boldsymbol{shell}}\left( \boldsymbol{t+dt} \right)\boldsymbol{>}\boldsymbol{r}_{\boldsymbol{shell}}^{\boldsymbol{MAX}}$

When the shell is growing beyond its maximal thickness, a portion of live cancer cells is transferred from the shell to the core to maintain $r_{shell}^{MAX}$ and the total tumour volume $V_{tumour}\left( t+dt \right)$, leading to an increase in core volume:

|  | $r_{core}\left( t+dt \right)=r_{tumour}\left( t+dt \right)-r_{shell}^{MAX}$ $V_{core}(t+dt)=\frac{4}{3}\pi{(r_{core}\left( t+dt \right))}^{3}$ | (S4.6) |
| --- | --- | --- |

The final volume of the proliferating shell is simply the difference between total and core volumes:

|  | $V_{shell}\left( t+dt \right)=V_{tumour}\left( t+dt \right)-V_{core}(t+dt)$ | (S4.7) |
| --- | --- | --- |

- **If** $\boldsymbol{r}_{\boldsymbol{shell}}\left( \boldsymbol{t+dt} \right)\boldsymbol{<}\boldsymbol{r}_{\boldsymbol{shell}}^{\boldsymbol{MAX}}$

When the shell is at least partially depleted, it is assumed that the core may shrink over time at maximal rate $s_{core}$. The rate of core shrinkage is impacted by the rate of depletion of the shell:

|  | $r_{core}\left( t+dt \right)= r_{core}\left( t+dt \right)*\left( 1-s_{core}\left( \frac{r_{shell}^{MAX}-r_{shell}\left( t+dt \right)}{r_{shell}^{MAX}} \right)*dt \right)$ | (S4.8) |
| --- | --- | --- |

The final core volume and total tumour volume are then updated as follows:

|  | $V_{core}(t+dt)=\frac{4}{3}\pi{(r_{core}\left( t+dt \right))}^{3}$ $V_{tumour}\left( t+dt \right)=V_{core}\left( t+dt \right)+V_{shell}\left( t+dt \right)$ | (S4.9) |
| --- | --- | --- |

- **Initialisation**

The total tumour volume $V_{tumor}\left( 0 \right)$ is initialised to the average tumour volume across animals at day 0 for each experimental arm. The shell is assumed to be at maximal thickness $r_{shell}\left( 0 \right)=r_{shell}^{MAX}$.

## **Cellular volume calculation in the outer tumour shell**

A sub-population *S* of cancer cells (see ***Supplementary Methods 3: Cell population PD model***) has a total cellular volume $V_{cells.pop}^{\left( S \right)}$. In the absence of cell death, this volume would double every $t_{d}$ hour, which can be expressed as an exponential growth from t=0 with initial volume $\Psi_{cell.0}$:

|  | $V_{cells.pop}^{\left( S \right)}(t)=\Psi_{cell.0}*e^{t*{ln(2)}/{t_{d}}}$ | (S5.1) |
| --- | --- | --- |

However, in the current DDR model not all cells will divide as they may die due to DNA damage, and thus Eq. S5.1 cannot be directly applied to compute the population cellular volume. Instead, this volume can be derived from the individual cell volume $V_{cell}^{\left( S \right)}$, which is identical for all cells in sub-population *S*, and the actual number of cancer cells $N_{cells}^{\left( S \right)}$ that remain in the pool (described in the next section ***Supplementary Methods 6: Cell number calculation***):

|  | $V_{cells.pop}^{\left( S \right)}\left( t+dt \right)=V_{cell}^{\left( S \right)}(t+dt)*N_{cells}^{\left( S \right)}(t+dt)$ | (S5.2) |
| --- | --- | --- |

The volume $V_{cell}^{\left( S \right)}$ of a single cell in sub-population *S* increases during the cell cycle from time 0 to time $t_{d}-dt$ following the equation S5.3 derived from Eq. S5.1 above:

|  | $V_{cell}^{\left( S \right)}\left( t+dt \right)=V_{cell}^{\left( S \right)}\left( t \right)*\left( 1+dt*\frac{\ln\left( 2 \right)}{t_{d}} \right)$ | (S5.3) |
| --- | --- | --- |

The total volume of cancer cells in the proliferating shell $V_{cells.shell}$ (Eq. S5.4) is the sum of the volumes of all the sub-populations (Eq. S5.2):

|  | $V_{cells.shell}\left( t+dt \right)=\sum_{S} V_{cells.pop}^{\left( S \right)}\left( t+dt \right)$ | (S5.4) |
| --- | --- | --- |

The relative change in $V_{cells.shell}$ defines the growth rate of the shell as shown in Eq. S4.2. Ultimately, only the relative change is considered and thus the initial volume $\Psi_{cell.0}$ can be set to an arbitrary value of 1.

## **Cell number calculation in the outer tumour shell**

The model tracks the number of live cancer cells $N_{cells}^{\left( S \right)}$ relative to time 0 in each sub-population *S* (see ***Supplementary Methods 3: Cell population PD model***), which is the sum of the cells normally proliferating $N_{cells.prolif}^{\left( S \right)}$ and the cells destined to delayed cell death $N_{cells.fated}^{\left( S \right)}$:

|  | $N_{cells}^{\left( S \right)}\left( t+dt \right)=N_{cells.prolif}^{\left( S \right)}\left( t+dt \right)+N_{cells.fated}^{\left( S \right)}\left( t+dt \right)$ | (S6.1) |
| --- | --- | --- |

DNA damage may cause normally proliferating cells to undergo either immediate cell death at rate $R_{kill}\left( t \right)$ (Eq. 6 in main text) or delayed cell death at rate $R_{delay\_kill}\left( t \right)$ (Eq. 7 in main text). In the latter case, the cells are transferred to the fated population. Therefore, the remaining number of normally proliferating cells at time $t+dt$ is:

|  | $N_{cells.prolif}^{\left( S \right)}\left( t+dt \right)=N_{cells.prolif}^{\left( S \right)}\left( t \right)*\left( 1-dt*R_{kill}\left( t+dt \right)-dt*R_{delay\_kill}(t+dt) \right)$ | (S6.2) |
| --- | --- | --- |

The cells that were transferred to the fated population (generation 0) lose their ability to form a colony as they may only proliferate up to seven divisions (generations 1 to 7), and their progeny ultimately die or become senescent, with increasing likelihood at every generation. At any time, the total number of fated cells in sub-population *S* is the sum of all generations and the senescent cells:

|  | $N_{cells.fated}^{\left( S \right)}\left( t \right)= N_{cells.fated.senesc}^{\left( S \right)}\left( t \right)+ \sum_{Y=0}^{7} N_{cells.fated.genY}^{\left( S \right)}\left( t \right)$ | (S6.3) |
| --- | --- | --- |

- **Throughout the cell cycle**

Until sub-population *S* reaches mitosis, the number of newly fated cells (generation 0) grows as a proportion of normally proliferating cells are transferred to this pool:

|  | $N_{cells.fated.gen0}^{\left( S \right)}\left( t+dt \right)=N_{cells.fated.gen0}^{\left( S \right)}\left( t \right)+ dt*R_{delay\_kill}\left( t+dt \right)*N_{cells.prolif}^{\left( S \right)}\left( t \right)$ | (S6.4) |
| --- | --- | --- |

The daughter fated cells in generations 1 to 7 die at a baseline rate $r_{kill.fated}$, which is increased by DSB damage-induced cell death $R_{kill}\left( t \right)$ (Eq. 6 in main text). The evolution of the number of cells in fated generation $Y=1,..,7$ is described by:

|  | $\underset{Y=1..7}{N_{cells.fated.genY}^{\left( S \right)}} \left( t+dt \right)=\underset{Y=1..7}{N_{cells.fated.genY}^{\left( S \right)}} \left( t \right)*\left( 1-dt*\alpha_{Y}*\left( r_{kill.fated}+R_{kill}\left( t+dt \right) \right) \right)$ | (S6.5) |
| --- | --- | --- |

where $\alpha_{Y}=0.2*Y (Y=1,..,7)$ is a generation-specific scaling factor to capture that each generation is more likely to undergo cell death as the cells accumulate more damage with each division.

The senescent cells stopped dividing but remain viable until they undergo cell death at constant rate $r_{kill.fated}$:

|  | $N_{cells.fated.senesc}^{\left( S \right)}\left( t+dt \right)=N_{cells.fated.senesc}^{\left( S \right)} \left( t \right)*\left( 1-dt*r_{kill.fated} \right)$ | (S6.6) |
| --- | --- | --- |

- **At mitosis**

The surviving normally proliferating cells replicate:

|  | $N_{cells.prolif}^{\left( S \right)}\left( t+dt \right)=2*N_{cells.prolif}^{\left( S \right)}\left( t+dt \right)$ | (S6.7) |
| --- | --- | --- |

The surviving cells in fated generation 0 to 7 (Eq. S6.4 and S6.5) can either divide and move up one generation with decreasing probability $\beta_{Y=0..7}\in\left[ 0,1 \right]$, or enter a senescent state with increasing probability ${1-\beta}_{Y=0..7}$:

|  | $\underset{Y=1..7}{N_{cells.fated.genY}^{\left( S \right)}} \left( t+dt \right)=2*\beta_{Y-1}*\underset{Y-1=0..6}{N_{cells.fated.genY}^{\left( S \right)}} \left( t+dt \right)$ | (S6.8) |
| --- | --- | --- |
|  | $N_{cells.fated.senesc}^{\left( S \right)}\left( t+dt \right)=N_{cells.fated.senesc}^{\left( S \right)}\left( t+dt \right)$  $+\sum_{Y=0}^{7} \left( 1-\beta_{Y} \right)*N_{cells.fated.genY}^{\left( S \right)}\left( t+dt \right)$ | (S6.9) |

where $\beta_{Y=0}=1$ since all fated cells in generation 0 will replicate and start their delayed cell death course (generation 1), and $\beta_{Y=7}=0$ since the cells stop their proliferation as they reached the maximum of possible divisions.

The pool of cells in fated generation 0 is accordingly reset to zero for the next cell cycle:

|  | $N_{cells.fated.gen0}^{\left( S \right)}\left( t+dt \right)=0$ | (S6.10) |
| --- | --- | --- |

- **Cells transfer from shell to inner core**

As the tumour proliferating layer grows, a portion of cancer cells may be transferred from this layer to the inner core to maintain the maximum thickness of the shell (see ***Supplementary Methods 4: Tumour volume calculation***). The number of cells is adjusted by backtracking from the actual relative change of shell volume, assuming that the proportion of each cell subgroup relative to the total number of cells ($\sum_{S} N_{cells}^{\left( S \right)}\left( t \right)$) is preserved.

Note that in practice this step is not mandatory as only relative changes in population size drive the change in tumour volume.

## **Pharmacokinetic models**

Compartmental pharmacokinetic (PK) models were used to compute drug plasma concentration over time after oral (PO) administration of the drugs. Parameters for all PK models are given in Supplementary Information. A two-compartment PK model was fitted to gartisertib plasma-concentration measurements taken in H2D Rag2 mice, including one IV profile (Table S1). The PK model was established using Phoenix WinNonlin 6.4 and NLME 1.2, with the FOCE algorithm and a multiplicative/proportional error model. Rucaparib PK was represented by a model with a central compartment and two transit compartments for absorption. The model was fitted to plasma-concentration measurements taken in non-tumour bearing NOD/SCID mice following single PO administration of rucaparib camsylate (Table S2; data source: [FDA report NDA 209115](https://www.accessdata.fda.gov/drugsatfda_docs/nda/2016/209115Orig1s000TOC.cfm); [*Multi-Discipline Review/Summary, Clinical, Non-Clinical*](https://www.accessdata.fda.gov/drugsatfda_docs/nda/2016/209115Orig1s000MultiDisciplineR.pdf)). The PK model was established in R using *minpack.lm* package and a multiplicative/proportional error model. Talazoparib PK was taken from the literature (15) and was represented by a one-compartment model (Table S3). All PK models were implemented in R to generate the drugs concentration profiles over time.

## **Preclinical TGI data**

TGI time series were collected from female, athymic nude-Foxn1 nu mice inoculated with TNBC PDXs (Table 2 in Main Text). These studies were performed by XenTech (Evry, France) in accordance with French legislation concerning the protection of laboratory animals and in accordance with a license approved by the French Ministry of Higher Education, Research and Innovation (registration numbers APAFIS#7125-2016012713169445v3, APAFIS#11191v2; APAFIS#19620-2019021311503803v1). Experimental procedures were reported earlier (16). The panel of tumour models included *BRCA* mutant (HBCx-10, HBCx-17, HBCx-22), *BRCA* wild-type HRD positive (HBCx-1, HBCx-9, HBCx-15) and HRD negative (HBCx-30, HBCx-33, T311R) PDXs. Age of animals at start of treatment for each experiment, in weeks: 10 (HBCx-15); 11 (HBCx-30, T311R); 12 (HBCx-9 study 4 and PDX panel, HBCx-17); 13 (HBCx-9 studies 1 and 3, HBCx-1, HBCx-10); 14 (HBCx-9 study 2, HBCx-22); 15 (HBCx-33). Mice were treated with either vehicle, PARP inhibitor monotherapy (rucaparib or talazoparib), ATR inhibitor monotherapy (gartisertib), or PARP + ATR inhibitor combination therapies, according to several dosing regimens, as listed in Table 2. For the PDX panel, gartisertib was given twice per week, x4 cycles, at doses of 10 mg/kg (HBCx-10, HBCx-17, HBCx-22, HBCx-1, HBCx-33) or 20 mg/kg (HBCx-9, HBCx-15, HBCx-30, T311R). All drugs were given PO. In all studies, treatment started at Day 1, except for the study in HBCx-1, where dosing started at Day 0. Other deviations included: in study 4, two-day dosing break (Day 9 and 10); study in HBCx-10, gartisertib dose at Day 4 was 10 mg/kg instead of 20 mg/kg; study in T311R, gartisertib dose at Day 22 was 10 mg/kg instead of 20 mg/kg. When dosing twice per day, the drugs were given 8 h apart.

# **Supplementary Tables**

## **Table S1. PK model parameters for gartisertib**

Model: two-compartment model with absorption.

| **Parameter** | **Value** | **CV%** |
| --- | --- | --- |
| Rate of absorption | *k_a_* = 0.46 /h | 0 |
| Central clearance | *CL* = 1.209 L/h/kg | 4.8 |
| Central volume of distribution | *V_c_* = 1.541 L/kg | 22.8 |
| Intercompartmental clearance | *Q* = 0.82 L/h/kg | 12.5 |
| Peripheral volume of distribution | *V_p_* = 8.935 L/kg | 29.2 |
| Bioavailability | *F_1_* = 38% | 12.5 |

## **Table S2. PK model parameters for rucaparib camsylate**

Model: one-compartment model with two transit compartments. Constraints: ka=ktr; Vc/F=1. Apparent PK parameters (bioavailability F not fitted).

| **Parameter** | **Value** | **CV%** |
| --- | --- | --- |
| Rate of absorption | *k_a_* = 0.54 /h | 12 |
| Transfer rate from 2^nd^ transit cmpt to central cmpt | *k_tr_* = 0.54 /h | - |
| Central clearance | *CL/F* = 6.6 L/h/kg | 14.5 |
| Central volume of distribution | *V_c_/F* = 1 L/kg | - |

## **Table S3. PK model parameters for talazoparib**

Model: one-compartment model with absorption, from Stewart et al 2014 (15). Apparent PK parameters (bioavailability F not fitted).

| **Parameter** | **Value** |
| --- | --- |
| Rate of absorption | *k_a_* = 5 /h |
| Central clearance | *CL/F* = 0.947 L/h/kg |
| Central volume of distribution | *V_c_/F* = 4.12 L/kg |

## **Table S4. Phase-specific processes during the cell cycle**

| G1 | S | G2 | M |
| --- | --- | --- | --- |
| Cell DDR | | | |
| SSB generation  -  DNA damage repair  Cell death | SSB generation  Conversion SSB->DSB  DNA damage repair  Cell death | SSB generation  -  DNA damage repair  Cell death | -  -  -  Cell death |
| PARPi | | | |
| DNA repair inhibition | DNA repair inhibition | DNA repair inhibition | - |
| ATRi | | | |
| DSB repair inhibition  - | DNA repair inhibition  Checkpoint override | DSB repair inhibition  Checkpoint override | -  Checkpoint override |

## **Table S5. PD model parameters fixed across all simulations**

*DAU = arbitrary unit of DNA damage.*

| **Parameter** | **Notation in equations** | **Value** | **Units** | **Source / rationale** |
| --- | --- | --- | --- | --- |
| Number of cell  sub-populations | - | 5 | - | Minimum value beyond which no significant difference was observed in the results |
| Maximum thickness of outer layer with proliferating cells | $r_{shell}^{MAX}$ | 0.25 | mm | Plausible value taken from (2) |
| Rate of core shrinkage (when outer layer depletes) | $s_{core}$ | 0.005 | /h | To fit the dynamics of tumour regression |
| Length of G1 phase | - | 0.5*$t_{d}$ | h | Proportions in the range of typical rapidly proliferating eukaryotic cell (17) |
| Length of S phase | - | 0.3*$t_{d}$ | h |  |
| Length of G2 phase | - | 0.15*$t_{d}$ | h |  |
| Length of M phase | - | 0.05*$t_{d}$ | h |  |
| Rate of endogenous generation of DSB damage | $r_{dsb}$ | 0 | DAU/h | The main endogenous source of DSBs is fork collapse triggered by SSBs during DNA replication, i.e. SSBs converted into DSBs during S phase (18) |
| Baseline rate of transformation of SSB into lethal DSB (S phase) | $r_{conv}$ | 0.03 | /h | To fit the TGI data |
| Baseline rate of SSB damage repair | ${rep}_{ssb}$ | 0.35 | /h | To fit the TGI data |
| Baseline rate of DSB damage repair | ${rep}_{dsb}$ | 0.1 | /h | To fit the TGI data, but slower than SSB repair |
| Baseline rate of damage-induced cell death | $r_{kill}$ | 3 | /DAU/h | To fit the TGI data |
| Rate of damage-induced cell death in G1 phase | $r_{kill}^{G1}$ | 0 | /DAU/h | To reflect that cells are more resistant to DNA damage in G1: more likely to arrest and repair rather than immediately undergo apoptosis |
| Rate of damage-induced cell death in S and G2 phases | $r_{kill}^{S,G2}$ | $r_{kill}$ | /DAU/h | To reflect that cells are vulnerable to lethal DNA damage due to the critical processes of DNA replication and preparation for mitosis |
| Rate of damage-induced cell death in M phase | $r_{kill}^{M}$ | 2.5*$r_{kill}$ | /DAU/h | To reflect mitotic catastrophe |
| Baseline rate of cell transfer towards delayed cell death in response to DNA damage | $r_{delay\_kill}$ | 4 | /DAU/h | To fit the TGI data, in particular the delay in tumour response |
| Baseline rate of cell death of fated cells | $r_{kill.fated}$ | 0.01 | /h | To fit the dynamics of tumour regression |

## **Table S6. Model parameters characterising the cancer cell line of the PDX panel.**

Table of cell specific DDR parameters for each PDX across the genetic background groups. For a full definition of the parameters see Table 1 in main. DAU = arbitrary unit of DNA damage. (*) HR for DSB repair only.

|  | **PDX** | **Cell doubling time** | **SSB endogenous damage** | **DNA damage repair** | | | |
| --- | --- | --- | --- | --- | --- | --- | --- |
|  |  |  |  | **Pathway PARP** | | **Pathway ATR/HR(*)** | |
|  |  |  |  | **SSB** | **DSB** | **SSB** | **DSB** |
|  |  | *t_doub_* | *r_ssb_* | *def_1_* | *def_3_* | *def_2_* | *def_4_* |
| BRCAm | HBCx-10 | 22 | 0.0005 | 0 | 0 | 0 | 85 % |
|  | HBCx-17 | 32 | 0.0005 | 0 | 0 | 0 | 95 % |
|  | HBCx-22 | 50 | 0.0005 | 0 | 0 | 0 | 35 % |
| BRCAwt HRD+ | HBCx-9 | 33 | 0.0005 | 0 | 0 | 0 | 50 % |
|  | HBCx-1 | 36 | 0.0005 | 0 | 0 | 0 | 40 % |
|  | HBCx-15 | 20 | 0.0005 | 0 | 85 % | 0 | 90 % |
| HRD- | HBCx-33 | 30 | 0.0005 | 0 | 0 | 0 | 0 |
|  | T311R | 36 | 0.0002 | 0 | 0 | 0 | 0 |
|  | HBCx-30 | 30 | 0.0002 | 0 | 0 | 0 | 0 |

# **Supplementary Figures**

## **Figure S1. Tumour growth models comparison.**

Fitting results for tumour growth models Mayneord and Simeoni. **A.** Observed tumour radius for untreated mice (grey lines and markers) from various experimental data sets with different PDX tumour models. Model simulations are overlaid with the data for different mathematical models of tumour growth: Mayneord, assuming linear growth of the radius (red), Simeoni, assuming first an exponential growth then linear growth of volume (yellow), and the model used in this paper, designated as “Virtual Tumour” (blue), which is based on the same principle as Mayneord. **B.** Precision of the fitted parameters across all 13 in-vivo experiments used in this paper (one parameter for Mayneord model, two parameters for Simeoni model). **C.** SSE = sum of squared errors of the fits.

**
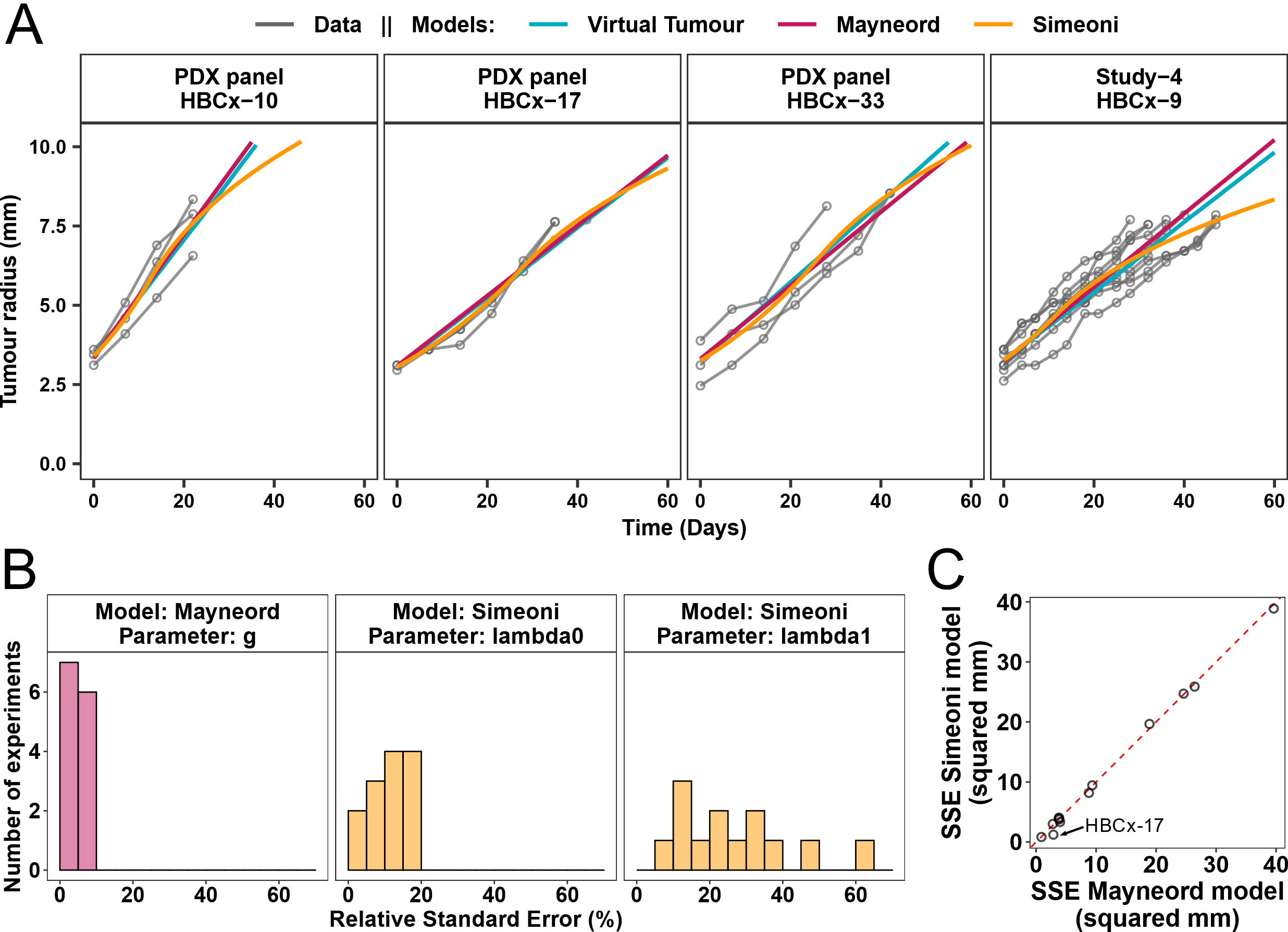
**

## **Figure S2. Model simulation: in the DDR model, three effects due to ATR inhibition contribute to the synergistic combination with the PARP inhibitor rucaparib.**

Simulation of a combination regimen rucaparib + gartisertib from study 1 in HBCx-9 tumour model (Figure 1B), in comparison with rucaparib monotherapy. Treatment starts at Day 1, last dosing at Day 35 (dotted line). Rucaparib is given twice (bid) a day x35 days at 100 mg/kg. Gartisertib is given once a day (qd) x35 days at 3 mg/kg. Combination simulations were run with either all ATR inhibitor drug effects or turning off one of the effects as specified.

**
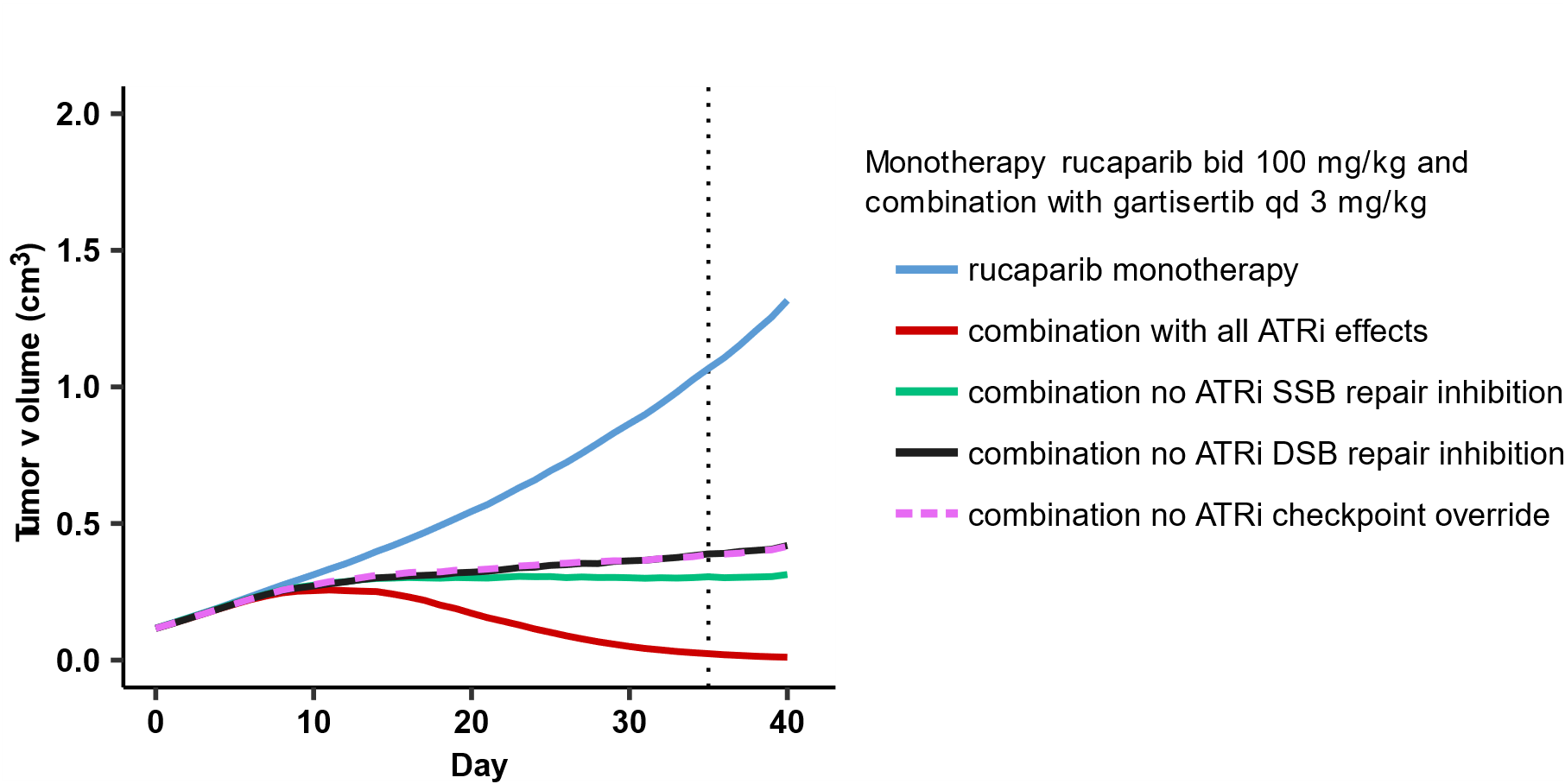
**

## **Figure S3. Model simulation overlaid with the TGI of each animal in experimental study 2 in HBCx-9 tumour model, and parameter local sensitivity.**

Coloured lines: animals’ data. Black lines: model simulation. Solid lines: default model simulations (parameters from Table 1 in main; cell doubling time $t_{doub}$ = 31h, HR deficiency parameter ${def}_{4}$=20%). Dotted and dashed lines: local sensitivity analysis, varying either **(A)** cell doubling time $t_{doub}$ = 24h (dotted line) and $t_{doub}$= 45h (dashed line) or **(B)** HR deficiency parameter ${def}_{4}$= 0% (dotted line), ${def}_{4}$= 60% (dot-dashed line) and ${def}_{4}$= 80% (dashed line).


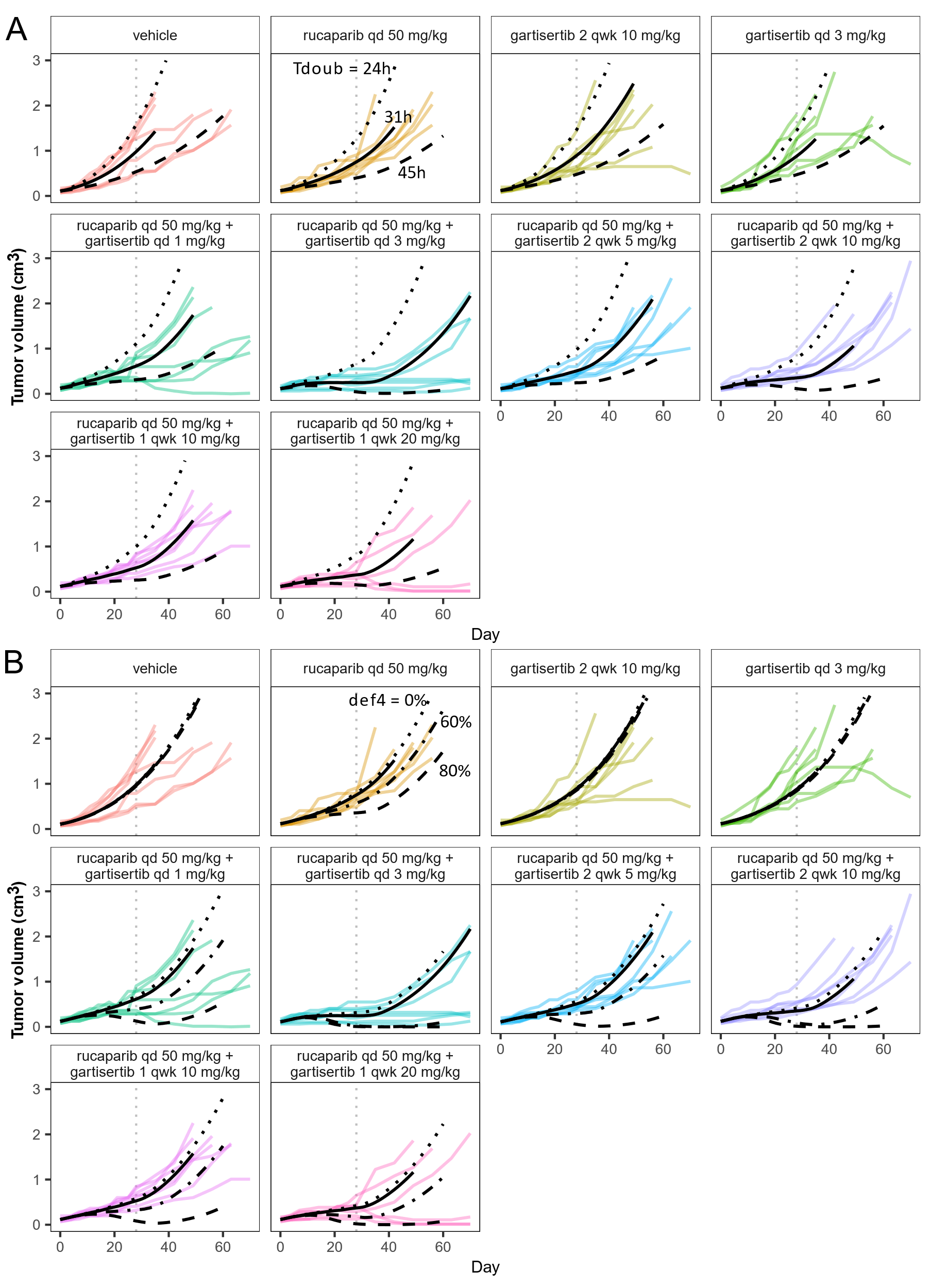


## **Figure S4. Fourth data set for the calibration of drug parameters. Model simulations overlaid with the TGI measured for various regimens in experimental study 4 in HBCx-9 tumour model.**

Treatment starts at Day 1, last dosing at Day 56 for talazoparib monotherapy (dot-dash line, panel A) and at Day 42 for all other regimens (dotted line). Solid line: simulation. Markers and error bars: data, mean tumour volume +/- SEM. Data for each arm are shown when at least 6 out of 9 mice are still in the experiment. In all PARPi treated arms talazoparib is given twice a day at 0.15 mg/kg. In all ATRi treated arms gartisertib is given once, twice or three times a week (1, 2, 3 qwk) x6 weeks at 20 mg/kg; for the arms with schedules 2 qwk and 3 qwk, dose was reduced to 10 mg/kg from Day 13. Protocol deviation also involved a two-days dosing break (Day 9 and 10).

**
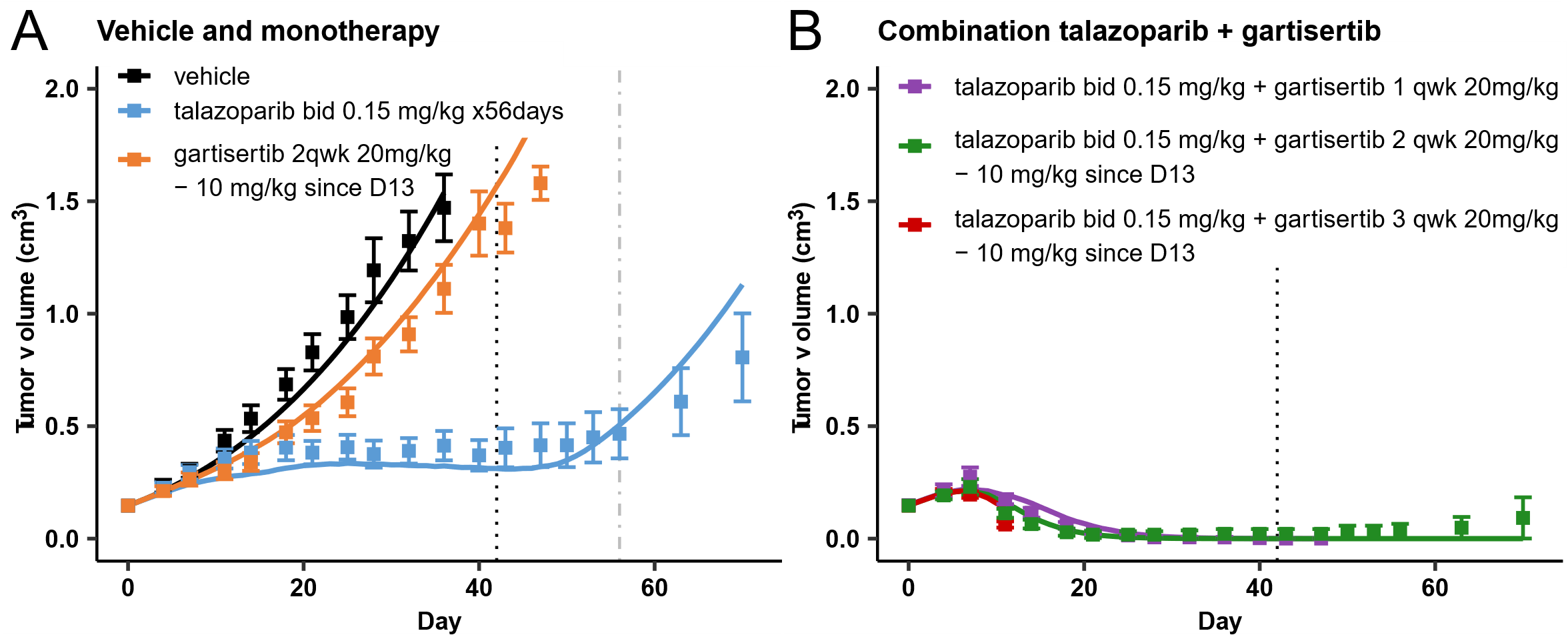
**

## **Figure S5. Simulated dose-exposure-response of rucaparib and talazoparib in monotherapy and in combination with gartisertib in HBCx-9.**

Model simulation of a range of doses, administered daily bid for 28 days, of rucaparib and talazoparib, in monotherapy and in combination with gartisertib daily qd 3 mg/kg. Simulations were conducted using the four parameter sets calibrated from the four studies in HBCx-9 (figures 2-4 and S4; specific parameters in Table 1 in main). The variables shown in each panel were measured on the last day of treatment (day 28), and then averaged (mean for tumour diameter, AUC for the other variables). Tumour diameter (panel B) and PD effects (panels C-G) were normalised to their respective values without treatment. The largest doses utilised in the PARPi monotherapy experiments are highlighted in panel A: 0.15 mg/kg bid and 100 mg/kg bid for talazoparib and rucaparib respectively.


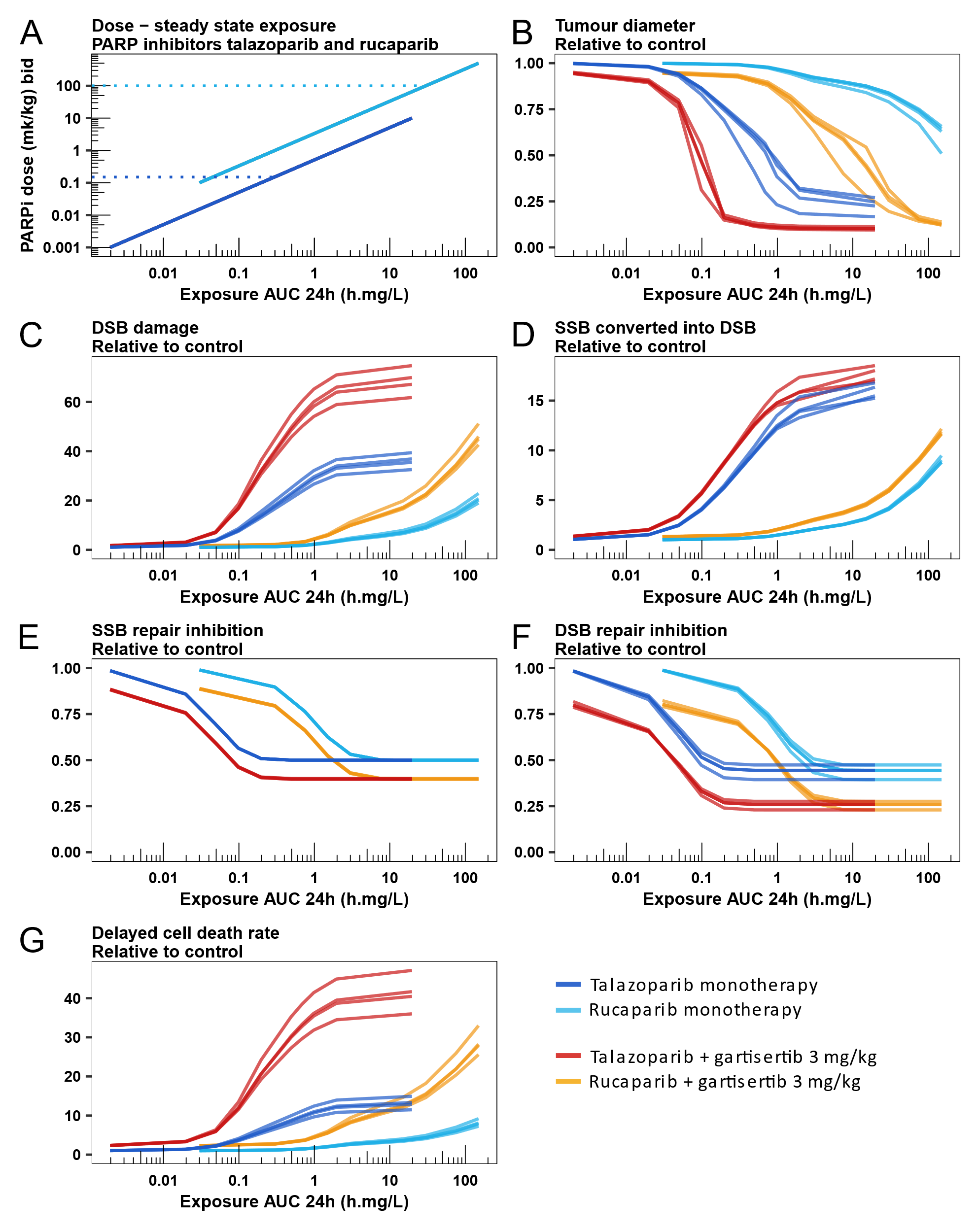


## **Figure S6. PDX panel (continued). Model simulations overlaid with the TGI data for the other studies in the TNBC PDX panel with different genetic backgrounds.**

Simulation (lines), data (black dots; n=3 animals per arm at start of treatment). The four arms in each study are split in columns. Each row is a different PDX tumour model with specific genetic background. **Treatments**. Gartisertib was given twice per week, PO, x4 cycles, at doses of 10 mg/kg (HBCx-10, HBCx-17, HBCx-22, HBCx-1, HBCx-33) or 20 mg/kg (HBCx-9, HBCx-15, HBCx-30, T311R). Talazoparib was given daily at 0.3 mg/kg qd x28. Treatment starts at Day 1, last dosing at Day 28.

**
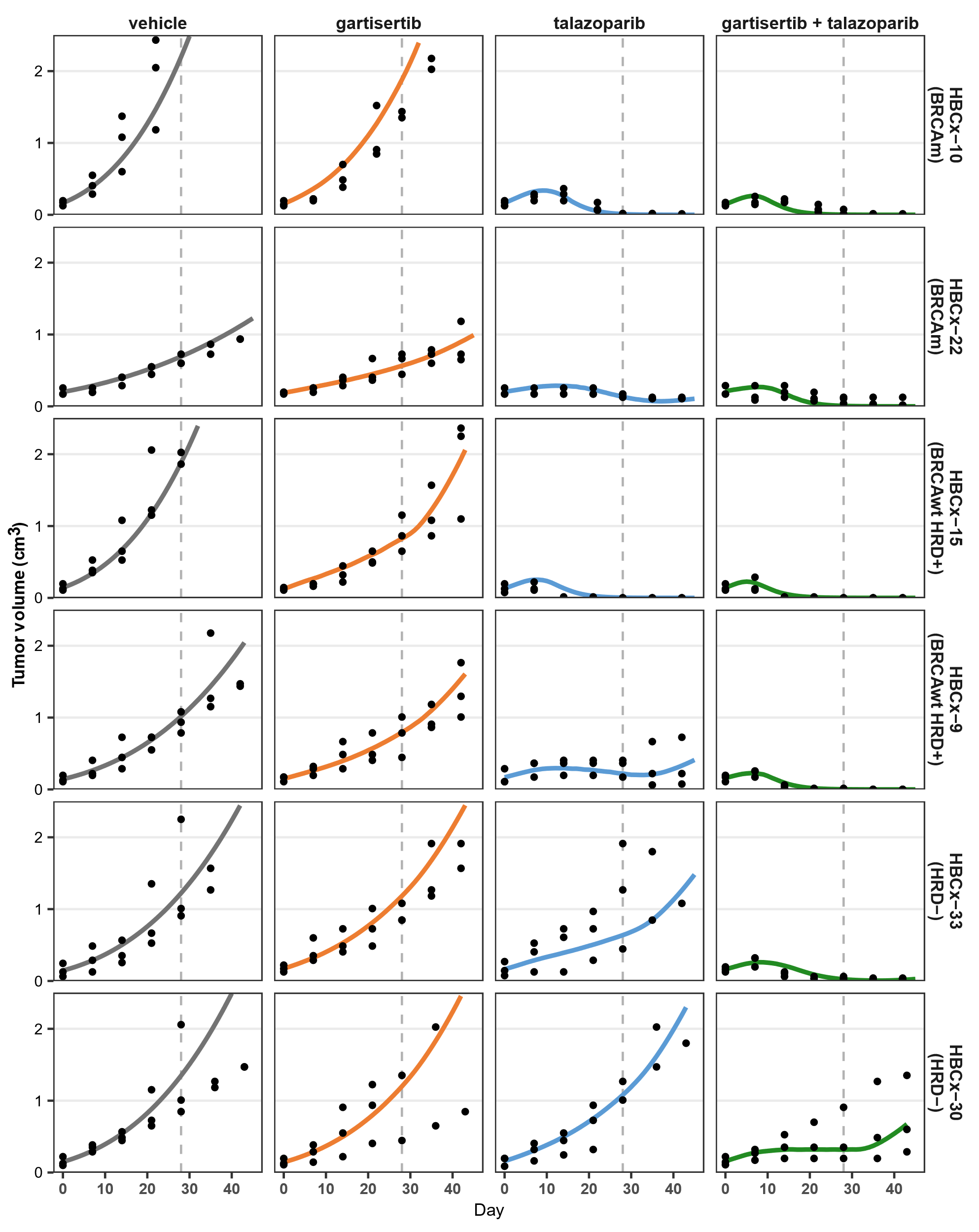
**

## **Figure S7. Preclinical PK model fitting for gartisertib.**

Oral dose except for the top left profile which is IV dose. D1 = day 1, D22 = day 22. Marker = data. Lines = model and CI.


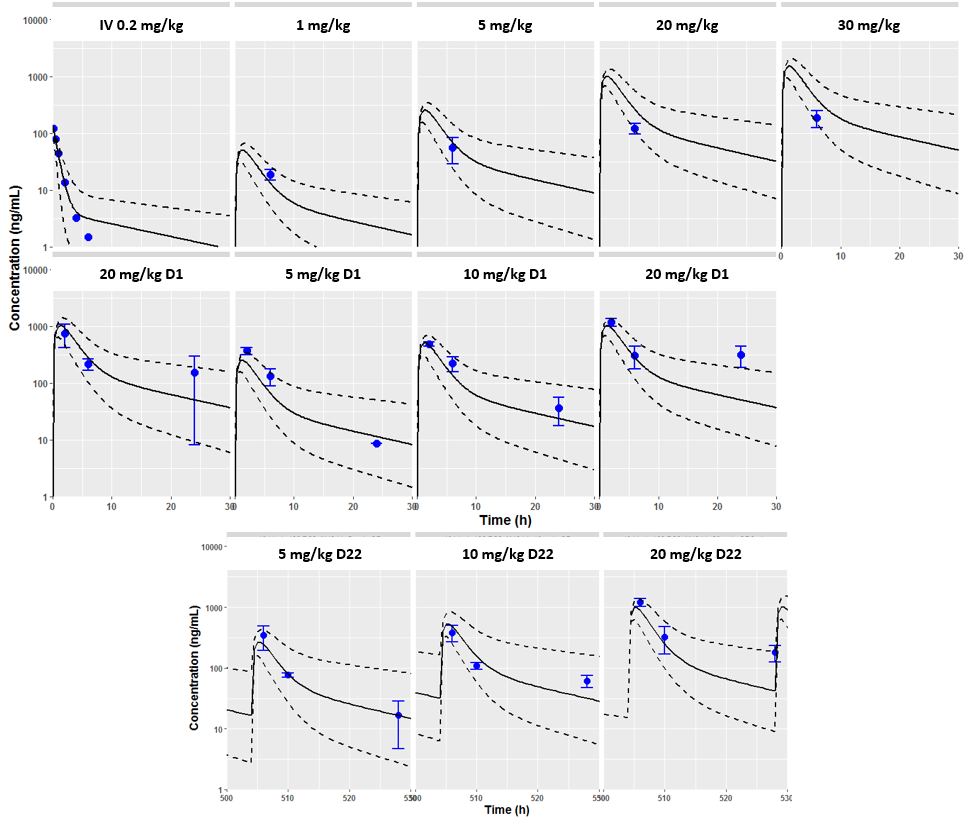


## **Figure S8. Preclinical PK model fitting for rucaparib.**

Dots = data. Lines = model.

**
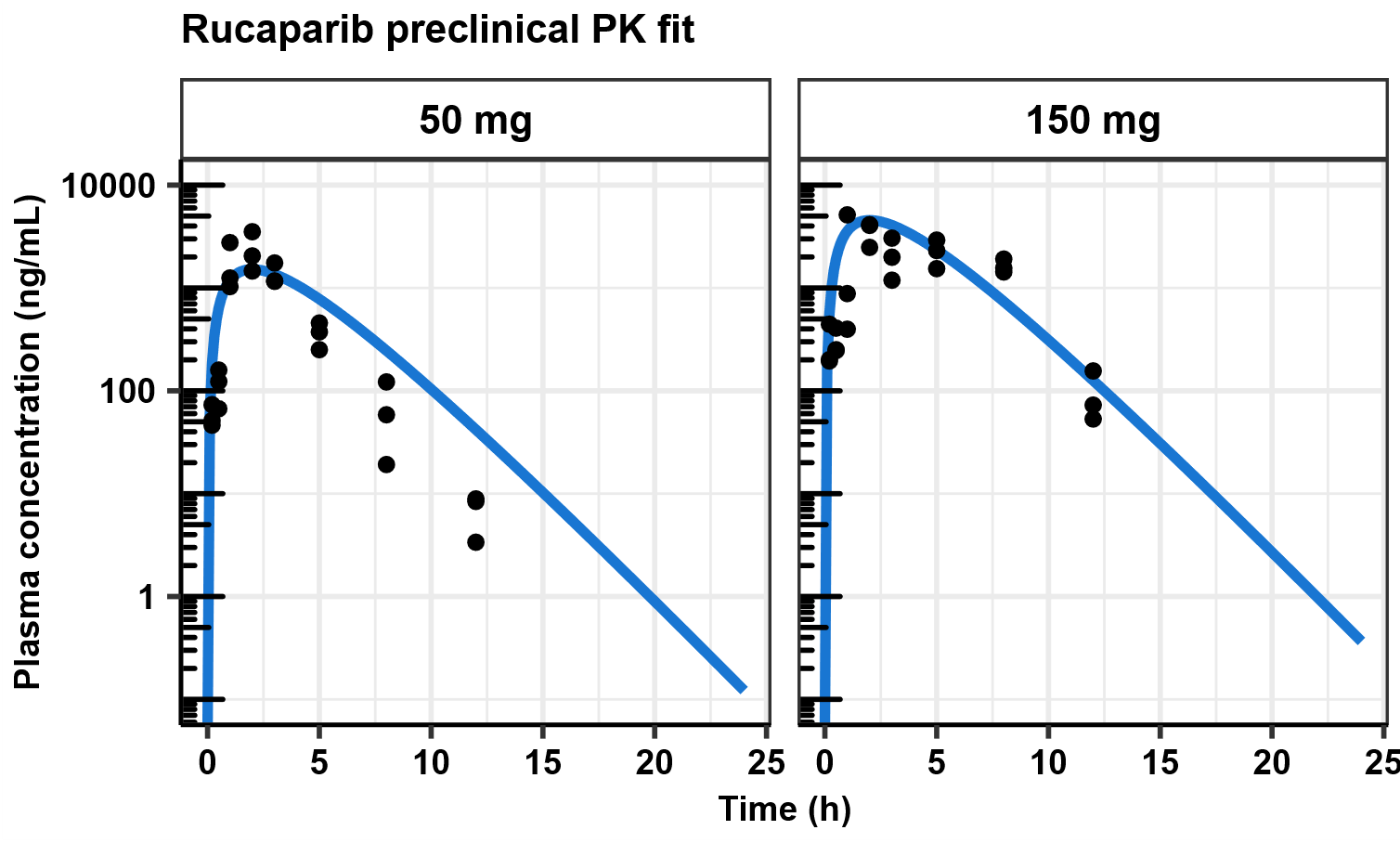
**

# **References**

1. Mayneord WV. On a Law of Growth of Jensen’s Rat Sarcoma. 1932;7.

2. Zaidi M, Fu F, Cojocari D, McKee TD, Wouters BG. Quantitative Visualization of Hypoxia and Proliferation Gradients Within Histological Tissue Sections. Front Bioeng Biotechnol. 2019 Dec 5;7:397.

3. Checkley S, MacCallum L, Yates J, Jasper P, Luo H, Tolsma J, et al. Bridging the gap between in vitro and in vivo: Dose and schedule predictions for the ATR inhibitor AZD6738. Sci Rep [Internet]. 2015 Oct [cited 2017 Nov 17];5(1). Available from: http://www.nature.com/articles/srep13545

4. Orrell D, Mistry HB. A simple model of a growing tumour. PeerJ. 2019 May 31;7:e6983.

5. Evans ND, Dimelow RJ, Yates JWT. Modelling of tumour growth and cytotoxic effect of docetaxel in xenografts. Comput Methods Programs Biomed. 2014 May;114(3):e3–13.

6. Yates JWT, Dudley P, Cheng J, D’Cruz C, Davies BR. Validation of a predictive modeling approach to demonstrate the relative efficacy of three different schedules of the AKT inhibitor AZD5363. Cancer Chemother Pharmacol. 2015 Aug;76(2):343–56.

7. Wang Y, Sung C, Dartois C, Ramchandani R, Booth BP, Rock E, et al. Elucidation of Relationship Between Tumor Size and Survival in Non-Small-Cell Lung Cancer Patients Can Aid Early Decision Making in Clinical Drug Development. Clin Pharmacol Ther. 2009 Aug;86(2):167–74.

8. Stein A, Wang W, Carter AA, Chiparus O, Hollaender N, Kim H, et al. Dynamic tumor modeling of the dose–response relationship for everolimus in metastatic renal cell carcinoma using data from the phase 3 RECORD-1 trial. BMC Cancer. 2012 Dec;12(1):311.

9. Simeoni M, Magni P, Cammia C, De Nicolao G, Croci V, Pesenti E, et al. Predictive pharmacokinetic-pharmacodynamic modeling of tumor growth kinetics in xenograft models after administration of anticancer agents. Cancer Res. 2004 Feb 1;64:1094–101.

10. Fernandez E, Mistry H, Brightman FA, Orrell D, Generali DG, Milani M, et al. From preclinical to phase II: Using virtual tumor clinical model to predict the efficacy of randomised phase II trials. J Clin Oncol. 2015 May 20;33(15_suppl):e12032–e12032.

11. Fernandez E, Orrell D, Brightman F, Fell D, Chassagnole C. Modeling ionizing radiation exposure in vitro and in vivo using the Virtual Tumour. Cancer Res. 2013 Apr 15;73(8 Supplement):5233–5233.

12. Ortega FG, Brightman FA, Orell D, Mistry H, Millen J, Chassagnole C. Abstract 680: Predicting the effect of radiotherapy on tumor growth inhibition and time to progression in head and neck cancer. In: Bioinformatics, Convergence Science, and Systems Biology [Internet]. American Association for Cancer Research; 2019 [cited 2020 Jan 21]. p. 680–680. Available from: http://cancerres.aacrjournals.org/lookup/doi/10.1158/1538-7445.AM2019-680

13. Cardilin T, Almquist J, Jirstrand M, Zimmermann A, Lignet F, El Bawab S, et al. Modeling long-term tumor growth and kill after combinations of radiation and radiosensitizing agents. Cancer Chemother Pharmacol. 2019 Jun;83(6):1159–73.

14. Forrester HB, Vidair CA, Albright N, Ling CC, Dewey WC. Using Computerized Video Time Lapse for Quantifying Cell Death of X-irradiated Rat Embryo Cells Transfected with c-myc or c-Ha-ras. Cancer Res. 1999;59:931–9.

15. Stewart E, Goshorn R, Bradley C, Griffiths LM, Benavente C, Twarog NR, et al. Targeting the DNA Repair Pathway in Ewing Sarcoma. Cell Rep. 2014 Nov;9(3):829–40.

16. Turchick A, Zimmermann A, Chiu LY, Dahmen H, Elenbaas B, Zenke FT, et al. Selective Inhibition of ATM-dependent Double-strand Break Repair and Checkpoint Control Synergistically Enhances the Efficacy of ATR Inhibitors. Mol Cancer Ther. 2023 Jul 5;22(7):859–72.

17. Cooper GM, Adams KW. The cell: a molecular approach. Ninth edition. New York, NY: Oxford University Press; 2023. 788 p.

18. Pfeiffer P. Mechanisms of DNA double-strand break repair and their potential to induce chromosomal aberrations. Mutagenesis. 2000 Jul 1;15(4):289–302.
